# Prediction of incident chronic kidney disease in community-based electronic health records: a systematic review and meta-analysis

**DOI:** 10.1093/ckj/sfae098

**Published:** 2024-04-18

**Authors:** Mohammad Haris, Keerthenan Raveendra, Christoforos K Travlos, Andrew Lewington, Jianhua Wu, Farag Shuweidhi, Ramesh Nadarajah, Chris P Gale

**Affiliations:** Leeds Institute for Cardiovascular and Metabolic Medicine, University of Leeds, Leeds, UK; Leeds Institute of Data Analytics, University of Leeds, Leeds, UK; Department of Cardiology, Leeds Teaching Hospitals NHS Trust, Leeds, UK; Faculty of Medicine and Health, University of Leeds, Leeds, UK; Faculty of Medicine, University of Patras, Greece; Renal Department, Leeds Teaching Hospitals NHS Trust, Leeds, UK; NIHR Leeds MedTech and In-Vitro Diagnostic Co-operative, Leeds, UK; Wolfson Institute of Population Health, Queen Mary University of London, London, UK; School of Dentistry, University of Leeds, Leeds, UK; Leeds Institute for Cardiovascular and Metabolic Medicine, University of Leeds, Leeds, UK; Leeds Institute of Data Analytics, University of Leeds, Leeds, UK; Department of Cardiology, Leeds Teaching Hospitals NHS Trust, Leeds, UK; Leeds Institute for Cardiovascular and Metabolic Medicine, University of Leeds, Leeds, UK; Leeds Institute of Data Analytics, University of Leeds, Leeds, UK; Department of Cardiology, Leeds Teaching Hospitals NHS Trust, Leeds, UK

**Keywords:** CKD, EHR, prediction models

## Abstract

**Background:**

Chronic kidney disease (CKD) is a major global health problem and its early identification would allow timely intervention to reduce complications. We performed a systematic review and meta-analysis of multivariable prediction models derived and/or validated in community-based electronic health records (EHRs) for the prediction of incident CKD in the community.

**Methods:**

Ovid Medline and Ovid Embase were searched for records from 1947 to 31 January 2024. Measures of discrimination were extracted and pooled by Bayesian meta-analysis, with heterogeneity assessed through a 95% prediction interval (PI). Risk of bias was assessed using Prediction model Risk Of Bias ASsessment Tool (PROBAST) and certainty in effect estimates by Grading of Recommendations, Assessment, Development and Evaluation (GRADE).

**Results:**

Seven studies met inclusion criteria, describing 12 prediction models, with two eligible for meta-analysis including 2 173 202 patients. The Chronic Kidney Disease Prognosis Consortium (CKD-PC) (summary c-statistic 0.847; 95% CI 0.827–0.867; 95% PI 0.780–0.905) and SCreening for Occult REnal Disease (SCORED) (summary c-statistic 0.811; 95% CI 0.691–0.926; 95% PI 0.514–0.992) models had good model discrimination performance. Risk of bias was high in 64% of models, and driven by the analysis domain. No model met eligibility for meta-analysis if studies at high risk of bias were excluded, and certainty of effect estimates was ‘low’. No clinical utility analyses or clinical impact studies were found for any of the models.

**Conclusions:**

Models derived and/or externally validated for prediction of incident CKD in community-based EHRs demonstrate good prediction performance, but assessment of clinical usefulness is limited by high risk of bias, low certainty of evidence and a lack of impact studies.

## Graphical Abstract

**Graphical Abstract:**
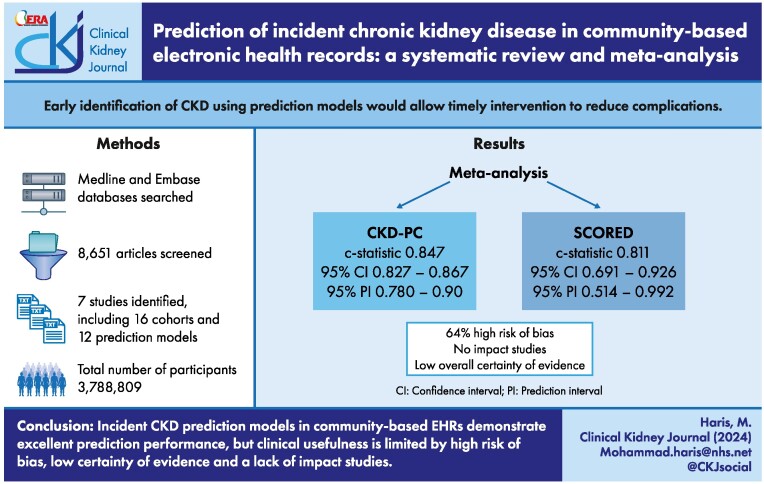

KEY LEARNING POINTS **What was known:**

- Chronic kidney disease (CKD) is a major global health problem, affecting over 800 million individuals worldwide.
- CKD carries substantial public health and economic implications.
- Timely interventions and novel treatments such as sodium-glucose cotransporter 2 inhibitors and finerenone can reduce the risk of disease progression and complications, but require early detection and diagnosis of CKD.

**This study adds:**

- Current models developed and/or validated in EHRs reveal good model discrimination performance.
- However, risk of bias is high in most studies, and certainty of effect estimates are low.
- Clinical utility is uncertain as there are no clinical utility analyses or clinical impact studies.

**Potential impact:**

- Prediction tools for incident CKD may help to reduce the health and wealth burden of the disease.
- Further work is required before they can be widely adopted in clinical practice.
- This includes investigating clinical utility, effects on future risk of CKD and complications, and cost benefit to a health system.

## INTRODUCTION

Chronic kidney disease (CKD) is a major global health problem affecting over 800 million individuals worldwide [1]. Its prevalence has increased partly due to rising incidences of diabetes mellitus (DM) and hypertension (HTN), and it is predicted to become the fifth leading cause of death worldwide by 2040 [2]. It also carries substantial public health and economic implications, annually costing the National Health Service £6.4 billion in the UK and Medicare $114 billion in the USA [3–5].

There is substantial interest in timely interventions and novel treatment options, such as sodium-glucose cotransporter 2 inhibitors and finerenone, which can reduce the risk of disease progression and cardiovascular complications [6–9]. However, REVEAL-CKD has shown that stage 3 CKD may be undiagnosed in up to 95% of patients [10]. Mass screening for CKD is controversial because of the potential costs involved [11, 12]. Current guidelines recommend screening individuals at risk of developing CKD according to a number of risk factors [13, 14], and the KDIGO Controversies Conference 2019 consensus recommends screening patients with risk factors and then using risk equations to guide the timing of subsequent testing [15].

A risk assessment tool to identify those at increased risk of reduced estimated glomerular filtration rate (eGFR) could facilitate screening for undiagnosed cases. The vast majority of the European population has a routinely collected electronic health record (EHR) in the primary care setting [16, 17]. A model that uses these data to risk stratify individuals for incident CKD could enable an effective and efficient targeted screening strategy. Previous research has shown that models developed in prospective cohorts may perform differently in EHRs [18]. In order to be applied to the general population through EHRs, models must be tested in EHRs or databases relevant to the general population or primary care (herein referred to as community-based EHRs).

Previous systematic reviews have either summarized models tested in prospective cohorts, where performance may not translate to community-based EHR data [19–21], or have included models predicting progression of CKD, which is not relevant to the initial identification of cases [20, 22]. To address this knowledge gap, we performed a systematic review and meta-analysis to identify prediction models for incident CKD derived and/or validated in community-based EHRs, and we synthesized discrimination performance of each model to identify which may be suitable to identify individuals at risk of CKD in clinical practice.

## MATERIALS AND METHODS

This systematic review has been reported in accordance with the Preferred Reporting Items for Systematic Reviews and Meta-Analyses (PRISMA) guidelines (Supplementary data).

### Search strategy and inclusion criteria

The Checklist for critical Appraisal and data extraction for systematic Reviews of prediction Modelling Studies (CHARMS) was used to frame the research question (Supplementary data). The Medline and Embase databases were searched through the Ovid platform from 1947 to 31 January 2024. A combination of keywords and subject headings related to CKD, prediction models and EHR were used. The search was restricted to the English language and to human studies. The full search strategy can be found in the Supplementary data. We manually performed forward and backward citation searches and looked through previous systematic reviews. We used Endnote's duplicate identification strategy and then manually removed all remaining duplicates.

Articles were included if they were an original study in human adults (≥18 years of age), developed and/or validated a prediction model(s) for incident CKD based on multivariable analysis in a community-based EHR, provided a prediction performance metric for discrimination performance and were written in English. Articles were excluded if they were prospective studies, included patients with CKD at baseline, only reported measures of association between risk factors and incident CKD rather than a full prediction model, studied only a subset of the general population (for example, individuals diagnosed with a particular morbidity) or incorporated variables that would not be routinely available in community-based EHR (e.g. C-cystatin, homocysteine levels, retinal photos; Supplementary data).

We uploaded records to a systematic review web application (Rayyan, Qatar Computing Research Institute) [23]. Three investigators (M.H., K.R. and C.K.T.) independently screened them for inclusion by title, abstract, full text and supplementary materials. Disagreements were resolved by consultation with a fourth investigator (R.N.).

### Data extraction and quality assessment

Three investigators (M.H., K.R. and C.K.T.) independently extracted the data from the included studies based on CHARMS. Discrepancies were resolved with a fourth investigator (R.N.). All data came from the primary reference, unless otherwise stated. We included data from derivation and external validation articles, including external validation data in community-based EHRs for models that were initially derived in prospective cohorts.

To allow quantitative synthesis and assessment of the predictive performance of the models we extracted measures of discrimination and calibration [24]. Discrimination assesses the model's ability to differentiate between individuals who will experience the outcome and those who will not. To assess discrimination, we extracted data on the c-statistic or the area under the receiver operating characteristic (AUROC), along with their corresponding 95% confidence intervals (95% CI). If the reported CI was missing, we computed it using the methods outlined by Debray *et al*. [24]. Calibration evaluates the accuracy of the model's predicted probabilities, and we extracted all performance measures reported. Three investigators (M.H., K.R. and C.K.T.) assessed the models for risk of bias and applicability to our review question using the Prediction model Risk Of Bias ASsessment Tool (PROBAST) [25].

We also checked for reporting of the clinical utility of a model (net benefit in the form of decision curve analysis or decision analytical modelling, which can be used to integrate the benefits and harms of using a model for clinical decision support) and conducted forward citation searching for studies determining the impact (clinical and cost-effectiveness) of using models in real world clinical practice.

### Data synthesis and statistical analysis

We reported continuous variables as means ± standard deviation and categorical variables as percentages. We evaluated statistical significance in all analyses at the 0.05 level. When a study reported on multiple cohorts and presented separate data for each cohort, we assessed model performance separately for each cohort within that study. A funnel plot was created to check for publication bias [26].

We conducted a Bayesian meta-analysis of discrimination through a summary measure of c-statistic and corresponding 95% CI. We calculated the 95% prediction interval (PI) to depict the extent of between-study heterogeneity and to indicate a possible range for prediction model performance in a new validation [27]. A prediction interval is a statistical measure to estimate a range for the predicted model performance in a new validation of the model with a certain level of confidence. Summary c-statistics of <0.60, 0.60–0.70, 0.70–0.80 and >0.80 were defined *a priori* as inadequate, adequate, acceptable and good based on prior publications [28, 29]. We conducted meta-analyses in R using the metafor and metamisc packages (R foundation for Statistical Computing 3.6.3) [30–32].

Our primary analysis assessed overall discrimination for models that had three or more cohorts with c-statistic data. We performed sensitivity analyses in which we restricted the primary analyses to only those studies where the participants domain in PROBAST assessment was ‘low’ risk of bias, and to only those studies where the overall PROBAST assessment was ‘low’ risk of bias. We performed a further sensitivity analysis where we excluded results from development and internal validation.

The Grading of Recommendations, Assessment, Development and Evaluation (GRADE) approach was used to assess the certainty of the evidence (Supplementary data). The certainty of the evidence was graded as high, moderate, low or very low [33].

## RESULTS

### Study selection

**Figure 1:**
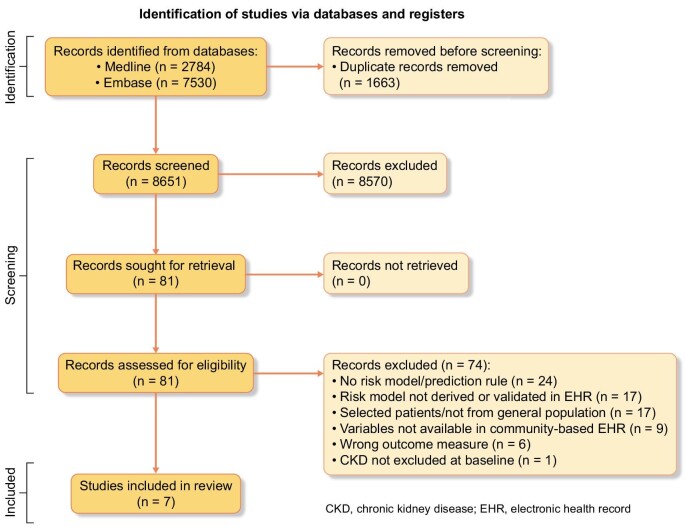
Flow diagram of literature search.

The study selection process is described in Fig. 1. We identified 7113 unique records, reviewed 81 full-text reports and included 7 studies. A list of excluded studies that met a number of the inclusion criteria is available in the Supplementary data.

### Characteristics of included studies

The 7 studies included 16 cohorts from a range of EHR databases located in USA (*n* = 11), Europe (*n* = 4) and Asia Pacific (*n* = 1) (Table 1). The total number of participants included in the studies was 3 788 809, with cohort sizes ranging from 2831 to 1 593 506. The mean age varied from 42.1 years to 59.6 years, and the proportion of women from 50% to 58%. Six studies used a definition of eGFR <60 mL/min/1.73 m^2^ for CKD, one study used eGFR <45 mL/min/1.73 m^2^ and one study did not clarify their definition (the authors were contacted but have not yet responded). Three studies used Chronic Kidney Disease Epidemiology Collaboration (CKD-EPI) calculation of eGFR, four studies used Modification of Diet in Renal Disease (MDRD) and one study did not clarify the equation used (the authors were contacted but have not yet responded).

**Table 1:** Baseline characteristics of included studies.

Study	Models	Cohort (country)	Study aim	EHR description	CKD cases (*n*)/total patients [*n* (%)]	Age (mean ± SD)	Female (%)	BMI (mean ± SD)	Diabetes (%)	Hypertension (%)	IHD/stroke (%)	Heart failure (%)	CVD (%)	Smoking (%)	Proteinuria (%)	PVD (%)	Kidney stones (%)	Outcome definition	Enrolment period (mean F/U in years)	Exclusion criteria
Nelson *et al.* 2019	CKD-PC	OptumLabs (US) 1–9	EV	Nationwide primary care	177 912/1 985 796 (8.96)	50 ± 16	58	29 ± 7.0	N/A	29	N/A	N/A	8	10	N/A	N/A	N/A	CKD (eGFR <60, CKD-EPI)	1970–2017 (4.2)	Missing values, mean follow-up >4 years
Stolpe *et al.* 2022	SCORED; Modified SCORED; Kearns; Kshirsagar; Kwon; Thakkinstian	HNR (Germany)	EV	State-mandated health services	360/4185 (8.60)	59.6 ± 7.8	50.5	27.8 ± 4.6	7.9	59.2	6.9	3.5	N/A	23.3	1.7	2.3	12	CKD (eGFR <60, CKD-Epi)	2000 (N/A)	Missing serum creatinine
Bang *et al.* 2007	SCORED	NHANES (US)	IV	Nationwide primary care	601/8530 (7.05)	46 ± 0.3	52	28 ± 0.1	8	34	N/A	2.1	4.9	20	10	2.7	N/A	CKD (eGFR <60, MDRD)	1999–2001 (N/A)	Missing serum creatinine or other covariates
Bang *et al.* 2007^b^	SCORED	ARIC (US)	EV	Nationwide primary care	392/12 038 (3.26)													CKD (eGFR <60, MDRD)	1987–89 (N/A)	Missing serum creatinine or other covariates
Fraccaro *et al.* 2016	SCORED; Chien; QKidney; Kshirsagar; Kwon; O'Seaghdha; Thakkinstian	SIRC (UK)	EV	Regional primary care	6038/162 653 (3.71)	42.1 ± 16.7	52.9	26.6 ± 6.0	N/A	N/A	N/A	N/A	N/A	51.1	0.5	N/A	N/A	CKD (eGFR <60, MDRD)	2009 (5)	CKD 3–5
Collins *et al.* 2012	QKidney	THIN (UK)	EV	Nationwide primary care	43 186/1 593 506 (2.71)	50 (median)	49.6	26.8 ± 4.7	3.7	9.3	N/A	0.6	5.3	53.2	N/A	1.3	0.3	CKD/ESRF (eGFR <45, MDRD)	2002–08	CKD at baseline
Shih *et al.* 2020^a^	Shih C4.5	Taiwan clinics (Taiwan)	D and IV	Nationwide primary care	5101/19 270 (26.47)	64.9 ± 11.5	58.1											CKD (not defined)	2015–19 (N/A)	
Perez-Monteoliva *et al.* 2015	HUGE formula	HERMEX (Spain)	EV	Regional primary care	62/2831 (2.19)	51.2 ± 14.7	53.5	28.6 ± 5.3	14.1	39.6	N/A	N/A	4.6	53.9	N/A	N/A	N/A	CKD (eGFR <60, CKD-EPI)	2007/2009 (N/A)	Non-residents, institutionalized and deceased persons, disabled subjects, pregnant women, people unable to give written informed consent

a In Yan and Shih, the baseline characteristics were reported separately for the CKD and non-CKD groups.

b In Bang the baseline characteristics were reported only for the derivation cohort and not the external validation cohort.

ARIC, Atherosclerosis Risk in Communities; BMI, body mass index; CVD, cerebrovascular disease; D, derivation; ESRF, end-stage renal failure; EV, external validation; FU, follow-up; HNR, Heinz-Nixdorf-Recall; IHD, ischaemic heart disease; IV, internal validation; NHANES, National Health and Nutrition Examination Surveys; PVD, peripheral vascular disease; SIRC, Salford Integrated Record cohort; THIN, The Health Improvement Network database.

### Characteristics of included prediction models

Twelve multivariable prediction models were derived and/or validated in EHRs. All studies reported the predictors used in the model. The longest prediction horizon was 5 years. Multivariable Cox or logistic regression were used in 11 models and machine learning techniques employed in 1 model. The optimum technique in the machine learning model was C4.5, chosen by discriminative performance (Supplementary data, Table S3).

**Figure 2:**
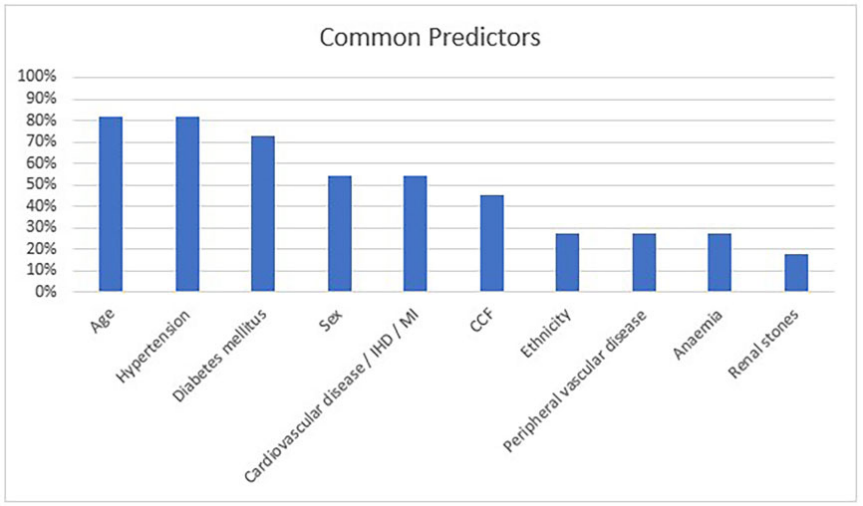
An overview of the ten predictors most frequently incorporated in the prediction models in this study. CCF, congestive cardiac failure; IHD, ischaemic heart disease; MI, myocardial infarction.

Supplementary data, Table S5 details the predictors used in each regression model. The most common predictors were age (82%), HTN (82%), DM (73%), sex (55%) and cardiovascular disease (55%), as shown in Fig. 2. The machine learning model only used demographic and diagnostic variables, as shown in Supplementary data, Table S6. Nine models had a c-statistic >0.8 on external validation. These were: Chronic Kidney Disease Prognosis Consortium (CKD-PC) (c-statistic >0.8 on 9 validations), SCreening for Occult REnal Disease (SCORED) (c-statistic >0.8 on 2 validations), QKidney (c-statistic >0.8 on 2 validation), Chien (c-statistic >0.8 on 1 validation), Kshirsagar (c-statistic >0.8 on 1 validation), Kwon (c-statistic >0.8 on 1 validation), O'Seaghdha (c-statistic >0.8 on 1 validation) and Thakkinstian (c-statistic >0.8 on 1 validation). Only six models (CKD-PC, SCORED, Kshirsagar, Kwon, Thakkinstian and QKidney) were externally validated in more than one cohort and only four studies reported calibration data.

### Clinical utility and clinical impact of included models

None of the included studies conducted a clinical utility analysis, and forward citation searching did not find any studies of clinical impact for included risk prediction models.

### Risk of bias assessment

**Figure 3:**
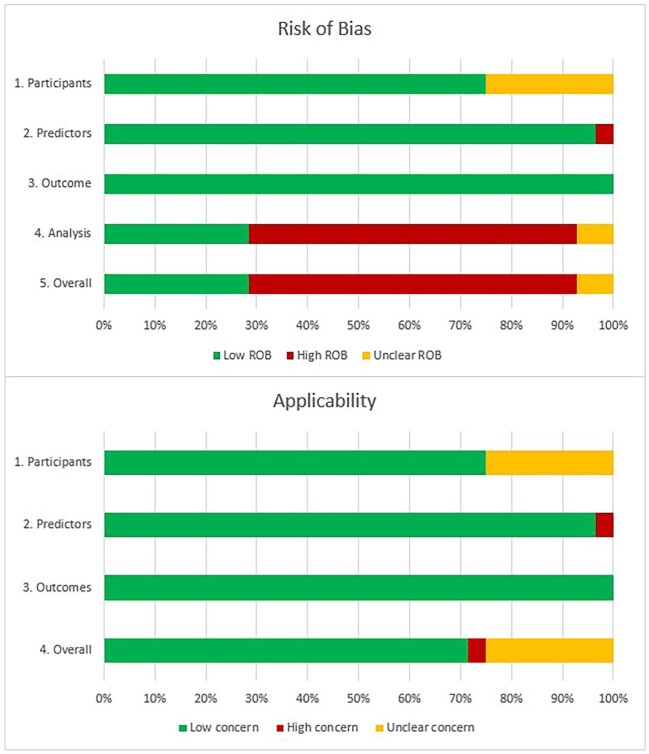
Judgements on the four PROBAST risk of bias domains and three PROBAST applicability domains presented as percentages across all included studies.

Supplementary data, Table S7 shows the results of the risk of bias and applicability assessment for each PROBAST domain for each model in the included studies. Figure 3 gives an overall summary of PROBAST domain assessments across all included studies. Overall, 63% of model results were at high risk of bias solely driven by high risk of bias in the analysis domain, mainly due to the handling of missing data in 56%.

### Meta analysis

**Figure 4:**
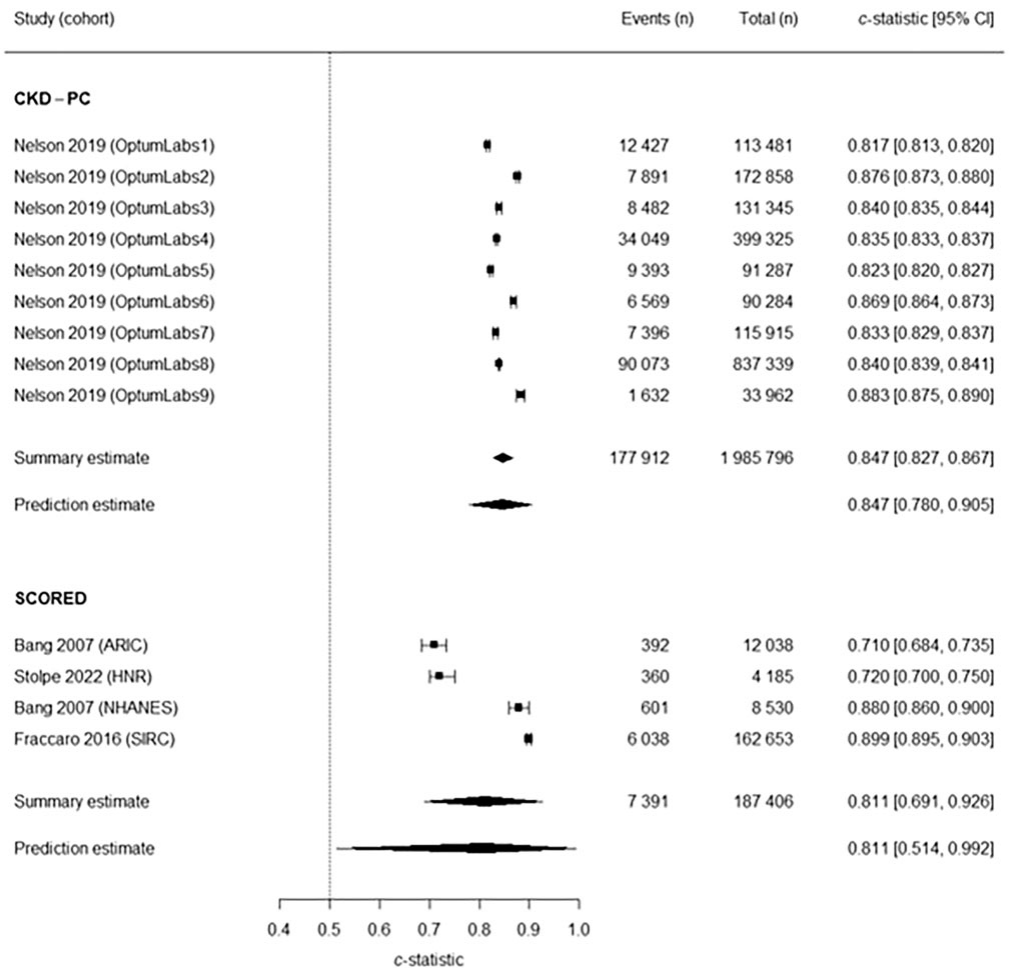
Forest plot of primary analysis of c*-*statistics. ARIC, Atherosclerosis Risk in Communities; HNR, Heinz-Nixdorf-Recall; NHANES, National Health and Nutrition Examination Surveys; SIRC, Salford Integrated Record cohort.

Two models (CKD-PC and SCORED) were eligible for the primary meta-analysis, incorporating 2173 202 patients. Both models’ results had good discrimination performance: CKD-PC (summary c-statistic 0.847; 95% CI 0.827–0.867; 95% PI 0.780–0.905; *n* = 9 cohorts; *n* = 1 985 796) and SCORED (summary c-statistic 0.811; 95% CI 0.691–0.926; 95% PI 0.514–0.992; *n* = 4 studies; *n* = 187 406), with wide heterogeneity evident for SCORED (Fig. 4).

When restricting the primary analysis to the three studies at low risk of bias for the participants domain of PROBAST, both the CKD-PC and SCORED models continued to demonstrate good prediction performance (Supplementary data, Fig. S1). After excluding results from development and internal validation, the SCORED model showed reduced prediction performance (Supplementary data, Fig. S2). No models were eligible for inclusion in analysis when excluding studies at overall high risk of bias. Funnel plots were symmetrical but with additional horizontal scatter (Supplementary data, Fig. S3), consistent with the presence of between-study heterogeneity.

### Certainty of evidence

The initial certainty level of the included prediction modelling studies was set at high because the association between the predictors and outcomes was considered irrespective of any causal connection. The overall certainty level was, however, downgraded to moderate, then low because of inconsistent results given high heterogeneity and the high overall risk of bias in included studies. The final overall certainty of evidence was low, implying that our confidence in the effect estimates of prediction model performance is limited and further research is very likely to change the effect estimate.

## DISCUSSION

This systematic review and meta-analysis included 12 models developed and/or validated in community-based EHR for estimating the risk of CKD. The majority of models showed good discrimination performance when externally validated in a community-based EHR. Two models (CKD-PC and SCORED) were eligible for primary meta-analysis with both demonstrating good summary discrimination performance measures. After excluding studies with overall high risk of bias, no model met eligibility criteria for meta-analysis. Clinical utility remains uncertain as none of the models underwent prospective investigation of clinical or cost-effectiveness.

### Clinical relevance

Multiple randomized controlled trials have demonstrated that novel treatment options and appropriate management of risk factors reduce disease progression and mortality for patients with CKD [34–36]. There is wide interest in how to ensure CKD cases are identified early in the disease trajectory in order to enable the implementation of disease-modifying therapies. Guidelines recommend screening patients with risk factors [12–14], but this can be resource intensive [11]. Risk prediction models may enable a more refined approach to early detection. Models developed and/or validated in community-based EHR cohorts using data widely available in the community can be increasingly utilized in healthcare environments across the world given the growing adoption of EHRs [16, 17]. Models developed in prospective studies were excluded from this review and analysis as previous research has shown they may perform differently in EHRs [18].

Some models, such as QKidney and O'Seaghdha, showed promising performance but had limited external validation and were therefore not eligible for meta-analysis. This highlights the importance of extensive external validation to enable reliable assessments of performance [37]. The CKD-PC and SCORED models were both eligible for meta-analysis, on account of external validation in multiple cohorts, and showed good discrimination performance. To aid implementation, the CKD-PC tool is available as an online calculator facilitating clinical application [38]. However, it was validated in cohorts within the same nation and published in one study, and therefore it is difficult to comment on the applicability of results to other geographies. In the meta-analysis of the SCORED model there was a large prediction interval, suggesting there is a large variability in potential performance in a new validation.

Furthermore, there remains uncertainty regarding the feasibility of implementing currently available models. Both the CKD-PC and SCORED models utilize data that may not be widely available in a large proportion of asymptomatic community-dwelling individuals, such as albumin urine creatinine ratio and high-density lipoprotein cholesterol. Furthermore, a lack of impact studies reduces confidence in their applicability to the general population for identifying incident cases. This is especially important given the high risk of bias we observed regarding reported performance measures, and poor reporting of calibration performance. Further work is required to determine the scale at which multivariable models may be utilized in the general population, whether early interventions based on these tools reduce future risk of CKD and its complications, and whether they confer a cost benefit at the level of health system. A prospective randomized assessment is required to assess how many extra cases may be detected using this approach, and whether it leads to a difference in the rate of adverse outcomes.

Furthermore, existing models can be improved. Albuminuria is a component of CKD, but was included as a risk factor in four models. These models mainly used eGFR as a diagnostic test for CKD and newer models incorporating albuminuria may prove to be more accurate. Ethnicity is a significant risk factor but was only included in three models, which may be due to inconsistent coding in EHRs. There is a pressing demand to identify more precise CKD predictors applicable to different populations given the emergence of more effective medications with substantial potential economic benefit given the cost of dialysis and impact on quality of life and mortality.

### Previous work

Previous reviews have evaluated CKD prediction models but do not specifically address whether these have been applied in community-based electronic health records, where it is most likely they would be of use in routine clinical practice and where most cases of CKD locate [19, 20, 22]. This review specifically focused on investigating models applicable to use in EHRs because this is a widely available medium through which these scores could be implemented at scale. Others have summarized models only for specific groups of patients (such as those with type 2 DM) or that estimate risk of progression of CKD [21]. This review excluded such models to increase applicability to the general population and focus on new-onset CKD. Consistent with previous reviews, we found suboptimal conduct in model development and a failure to progress to impact studies [19–22].

### Strengths and limitations

We used a comprehensive search strategy to identify all relevant articles and models and performed a thorough analysis. We ensured applicability in primary care settings by only including models from community-based cohorts and those that incorporated variables readily available in such settings.

We acknowledge the limitations of our study. We restricted our search strategy to articles written in English, although this has not been shown to lead to significant bias [39]. Meta-analysis of calibration performance was not possible due to lack of calibration reporting. We did not present meta-regression or subgroup meta-analysis to investigate heterogeneity between studies based on study-level characteristics or subgroups in the absence of available individual patient data given that such analyses would be prone to ecological bias [40], and are inferior to subgroup results–derived patient-level data [24]. The funnel plot (Supplementary data, Fig. S3) shows significant horizontal scatter, demonstrating between-study heterogeneity. Between-study heterogeneity can occur due to differences in study characteristics, study quality or studied populations. Study populations varied in mean age, proportion who were women, comorbidity burden and the proportion of observed CKD cases. There is incomplete coding in community-based EHRs of potentially important variables that are thus not included in models. It is also possible that coding of CKD may be incomplete in community-based EHRs, so the incidence of CKD in the included studies may be underestimated. Missing data is a commonly observed shortfall in prediction modelling research [41], even in models recommended for use in healthcare [42]. Anaemia is included as a variable in models and anaemia is associated with CKD, but causality cannot be assumed as patients with anaemia may have latent undiagnosed CKD rather than go on to develop CKD.

## CONCLUSION

This systematic review and meta-analysis identified 12 risk prediction models for incident CKD developed and/or validated in community-based EHRs. The models showed variable prediction performance for incident CKD, but were limited due to high risk of bias, missing data, low certainty of evidence and a lack of impact studies. Therefore, the utility of these models in clinical practice remains undetermined.

## Supplementary Material

sfae098_Supplemental_File

## Data Availability

The data underlying this article are available in the article itself and the supplementary material.

## References

[1] Kovesdy CP. Epidemiology of chronic kidney disease: an update 2022. Kidney Int Suppl (2011) 2022;12:7–11. 10.1016/j.kisu.2021.11.003 35529086 PMC9073222

[2] Foreman KJ, Marquez N, Dolgert A et al. Forecasting life expectancy, years of life lost, and all-cause and cause-specific mortality for 250 causes of death: reference and alternative scenarios for 2016–40 for 195 countries and territories. Lancet 2018;392:2052–90. 10.1016/S0140-6736(18)31694-5 30340847 PMC6227505

[3] Kidney Research UK. Kidney Disease: A UK Public Health Emergency . The Health Economics of Kidney Disease to 2033. 2023 [Internet]. Available from: https://www.kidneyresearchuk.org/wp-content/uploads/2023/06/Economics-of-Kidney-Disease-full-report_accessible.pdf (21 January 2024, date last accessed).

[4] Couser WG, Remuzzi G, Mendis S et al. The contribution of chronic kidney disease to the global burden of major noncommunicable diseases. Kidney Int 2011;80:1258–70. 10.1038/ki.2011.368 21993585

[5] Saran R, Robinson B, Abbott KC et al. US Renal Data System 2018 Annual Data Report: epidemiology of kidney disease in the United States. Am J Kidney Dis 2019;73:A7–8. 10.1053/j.ajkd.2019.01.001 30798791 PMC6620109

[6] Martinez YV, Benett I, Lewington AJP et al. Chronic kidney disease: summary of updated NICE guidance. BMJ 2021;374:n1992. 10.1136/bmj.n1992 34489303

[7] Uhlig K, Levey AS. Developing guidelines for chronic kidney disease: we should include all of the outcomes. Ann Intern Med 2012;156:599–601. 10.7326/0003-4819-156-8-201204170-00012 22508736

[8] Levey AS, Coresh J. Chronic kidney disease. Lancet 2012;379:165–80. 10.1016/S0140-6736(11)60178-5 21840587

[9] Bakris GL, Agarwal R, Anker SD et al. Effect of finerenone on chronic kidney disease outcomes in type 2 diabetes. N Engl J Med 2020;383:2219–29. 10.1056/NEJMoa2025845 33264825

[10] Tangri N, Moriyama T, Schneider MP et al. Prevalence of undiagnosed stage 3 chronic kidney disease in France, Germany, Italy, Japan and the USA: results from the multinational observational REVEAL-CKD study. BMJ Open 2023;13:e067386. 10.1136/bmjopen-2022-067386 PMC10230905 37217263

[11] Tonelli M, Tiv S, Anand S et al. Diagnostic yield of population-based screening for chronic kidney disease in low-income, middle-income, and high-income countries. JAMA Netw Open 2021;4:e2127396. 10.1001/jamanetworkopen.2021.27396 34605917 PMC8491102

[12] Fink HA, Ishani A, Taylor BC et al. Screening for, monitoring, and treatment of chronic kidney disease stages 1 to 3: a systematic review for the U.S. Preventive Services Task Force and for an American College of Physicians Clinical Practice Guideline. Ann Intern Med 2012;156:570–81. 10.7326/0003-4819-156-8-201204170-00008 22508734

[13] National Institute for Health and Care Excellence. Chronic kidney disease: assessment and management (NICE guideline NG203) . 2021, https://www.nice.org.uk/guidance/ng203 (19 January 2024, date last accessed). 35077091

[14] Kidney Disease: Improving Global Outcomes (KDIGO) CKD Work Group. KDIGO clinical practice guideline for the evaluation and management of chronic kidney disease . Kidney Int Suppl 2013;3:1–150.

[15] Shlipak MG, Tummalapalli SL, Boulware LE et al. The case for early identification and intervention of chronic kidney disease: conclusions from a Kidney Disease: Improving Global Outcomes (KDIGO) Controversies Conference. Kidney Int 2021;99:34–47. 10.1016/j.kint.2020.10.012 33127436

[16] Chao TF, Liu CJ, Lin YJ et al. Oral anticoagulation in very elderly patients with atrial fibrillation: a nationwide cohort study. Circulation 2018;138:37–47. 10.1161/CIRCULATIONAHA.117.031658 29490992

[17] Schnabel RB, Sullivan LM, Levy D et al. Development of a risk score for atrial fibrillation (Framingham Heart Study): a community-based cohort study. Lancet 2009;373:739–45. 10.1016/S0140-6736(09)60443-8 19249635 PMC2764235

[18] Kolek MJ, Graves AJ, Xu M et al. Evaluation of a prediction model for the development of atrial fibrillation in a repository of electronic medical records. JAMA Cardiol 2016;1:1007–13. 10.1001/jamacardio.2016.3366 27732699 PMC5293184

[19] Collins GS, Omar O, Shanyinde M et al. A systematic review finds prediction models for chronic kidney disease were poorly reported and often developed using inappropriate methods. J Clin Epidemiol 2013;66:268–77. 10.1016/j.jclinepi.2012.06.020 23116690

[20] Echouffo-Tcheugui JB, Kengne AP. Risk models to predict chronic kidney disease and its progression: a systematic review. PLoS Med 2012;9:e1001344. 10.1371/journal.pmed.1001344 23185136 PMC3502517

[21] González-Rocha A. Risk prediction score for chronic kidney disease in healthy adults and adults with type 2 diabetes: systematic review. Prev Chronic Dis 2023;20:E30. 37079751 10.5888/pcd20.220380 PMC10159345

[22] Ramspek CL, de Jong Y, Dekker FW et al. Towards the best kidney failure prediction tool: a systematic review and selection aid. Nephrol Dial Transplant 2020;35:1527–38. 10.1093/ndt/gfz018 30830157 PMC7473808

[23] Ouzzani M, Hammady H, Fedorowicz Z et al. Rayyan—a web and mobile app for systematic reviews. Syst Rev 2016;5:210. 10.1186/s13643-016-0384-4 27919275 PMC5139140

[24] Debray TPA, Damen JAAG, Snell KIE et al. A guide to systematic review and meta-analysis of prediction model performance. BMJ 2017;356:i6460. 10.1136/bmj.i6460 28057641

[25] Moons KGM, Wolff RF, Riley RD et al. PROBAST: a tool to assess risk of bias and applicability of prediction model studies: explanation and elaboration. Ann Intern Med 2019;170:W1–33. 10.7326/M18-1377 30596876

[26] Bridge J, Blakey JD, Bonnett LJ. A systematic review of methodology used in the development of prediction models for future asthma exacerbation. BMC Med Res Method 2020;20:22. 10.1186/s12874-020-0913-7 PMC7003428 32024484

[27] Debray TP, Damen JA, Riley RD et al. A framework for meta-analysis of prediction model studies with binary and time-to-event outcomes. Stat Methods Med Res 2019;28:2768–86. 10.1177/0962280218785504 30032705 PMC6728752

[28] Lloyd-Jones DM. Cardiovascular risk prediction: basic concepts, current status, and future directions. Circulation 2010;121:1768–77. 10.1161/CIRCULATIONAHA.109.849166 20404268

[29] Khan SS, Ning H, Shah SJ et al. 10-Year risk equations for incident heart failure in the general population. J Am Coll Cardiol 2019;73:2388–97. 10.1016/j.jacc.2019.02.057 31097157 PMC6527121

[30] Riley RD, Tierney JF, Stewart LA, eds. Individual participant data meta-analysis: a handbook for healthcare research (Wiley series in statistics in practice). Hoboken, NJ: Wiley, 2021;1.

[31] R Core Team . R: a language and environment for statistical computing [Internet]. Available from: https://www.R-project.org/. Vienna, Austria: R Foundation for Statistical Computing, 2018.

[32] Viechtbauer W. Conducting meta-analyses in R with the metafor package. J Stat Softw 2010;36:1–48. 10.18637/jss.v036.i03

[33] Remoortel HV, Scheers H, Buck ED et al. . et al Prediction modelling studies for medical usage rates in mass gatherings: a systematic review. PLoS One 2020;15:e0234977. 10.1371/journal.pone.0234977 32574190 PMC7310685

[34] Heerspink HJL, Stefánsson BV, Correa-Rotter R et al. Dapagliflozin in patients with chronic kidney disease. N Engl J Med 2020;383:1436–46. 10.1056/NEJMoa2024816 32970396

[35] Neal B, Perkovic V, Mahaffey KW et al. Canagliflozin and cardiovascular and renal events in type 2 diabetes. N Engl J Med 2017;377:644–57. 10.1056/NEJMoa1611925 28605608

[36] EMPA-KIDNEY Collaborative Group; Herrington WG, Staplin N, Wanner C et al. Empagliflozin in patients with chronic kidney disease. N Engl J Med 2023;388:117–27. 10.1056/NEJMoa2204233 36331190 PMC7614055

[37] Ramspek CL, Jager KJ, Dekker FW et al. External validation of prognostic models: what, why, how, when and where? Clin Kidney J 2021;14:49–58. 10.1093/ckj/sfaa188 33564405 PMC7857818

[38] CKD Risk Tool. [Internet ] [cited 8 June 2023]. Available from: https://ckdpcrisk.org/ckdrisk/ (21 January 2024, date last accessed).

[39] Morrison A, Polisena J, Husereau D et al. The effect of English-language restriction on systematic review-based meta-analyses: a systematic review of empirical studies. Int J Technol Assess Health Care 2012;28:138–44. 10.1017/S0266462312000086 22559755

[40] Berlin JA, Santanna J, Schmid CH et al. Anti-Lymphocyte Antibody Induction Therapy Study Group. Individual patient- versus group-level data meta-regressions for the investigation of treatment effect modifiers: ecological bias rears its ugly head. Stat Med 2002;21:371–87. 10.1002/sim.1023 11813224

[41] Nijman S, Leeuwenberg AM, Beekers I et al. Missing data is poorly handled and reported in prediction model studies using machine learning: a literature review. J Clin Epidemiol 2022;142:218–29. 10.1016/j.jclinepi.2021.11.023 34798287

[42] Tsvetanova A, Sperrin M, Peek N et al. Missing data was handled inconsistently in UK prediction models: a review of method used. J Clin Epidemiol 2021;140:149–58. 10.1016/j.jclinepi.2021.09.008 34520847

