# Supplementary material for: Prediction of incident chronic kidney disease in community-based electronic health records: a systematic review and meta-analysis
Source: Clin Kidney J. 2024 Apr 18;17(5):sfae098. doi: 10.1093/ckj/sfae098 (PMC11087823; doi:10.1093/ckj/sfae098)
Supplement: sfae098_Supplemental_File [file sfae098_supplemental_file.docx]

**Supplementary Appendix**

Prediction of incident chronic kidney disease in community-based electronic health records: a systematic review and meta-analysis

[SUPPLEMENTARY METHODS 3](#_Toc154737812)

[Formulation of research question using CHARMS (CHecklist for critical Appraisal and data extraction for systematic Reviews of prediction Modelling Studies): 3](#_Toc154737813)

[Search Terms and search strategy with full results 4](#_Toc154737814)

[Inclusion and exclusion criteria pertaining to variables incorporated in models 7](#_Toc154737815)

[PROBAST: Justifications for assessments for specific signalling questions 8](#_Toc154737816)

[Excluded studies 9](#_Toc154737817)

[Bayesian meta-analysis of *c*-statistic / AUROC 12](#_Toc154737818)

[GRADE: Standards used for judging criteria for downgrading and upgrading certainty of evidence 13](#_Toc154737819)

[SUPPLEMENTARY RESULTS 14](#_Toc154737820)

[Studies included in systematic review and meta-analysis 14](#_Toc154737821)

[Supplementary Table S1: Outcomes of studies reporting on prediction models 15](#_Toc154737822)

[Supplementary Table S2: Calibration performance of models 16](#_Toc154737823)

[Supplementary Table S3: Performance of machine learning and traditional regression techniques during model development 17](#_Toc154737824)

[Supplementary Table S4: Cohorts and number of predictors 18](#_Toc154737825)

[Supplementary Table S5: Baseline variables used in regression models 19](#_Toc154737826)

[Supplementary Table S6: Baseline variable categories used in machine learning models 20](#_Toc154737827)

[Supplementary Table S7: Risk of bias and applicability assessment for each PROBAST domain 21](#_Toc154737828)

[Supplementary Figure S1: Sensitivity analysis for prediction models restricted to studies with low risk of bias for participants domain of PROBAST 23](#_Toc154737829)

[Supplementary Figure S2: Sensitivity analysis for prediction models excluding results from development and internal validation 24](#_Toc154737830)

[Supplementary Figure S3: Funnel plot 25](#_Toc154737831)

[PRISMA CHECKLIST 26](#_Toc154737832)

[PRISMA ABSTRACT CHECKLIST 29](#_Toc154737833)

[REFERENCES 30](#_Toc154737834)

# SUPPLEMENTARY METHODS

## Formulation of research question using CHARMS (CHecklist for critical Appraisal and data extraction for systematic Reviews of prediction Modelling Studies):

| **CHARMS key items to guide framing of review, search strategy and study inclusion and exclusion criteria** | **Comments for this systematic review** |
| --- | --- |
| Prognostic versus diagnostic prediction model | Prognostic prediction model |
| Intended scope of the review | Models to inform referral for diagnostic testing |
| Types of Prediction modelling studies | Prediction model development without external validation in independent data, prediction model development with external validation in independent data, external model validation, possibly with model updating |
| Target population to whom the prediction model applies | Adults in the general population |
| Outcome to be predicted | Specific future event, diagnosis of chronic kidney disease |
| Time span of prediction | Any time interval |
| Intended moment of using the model | Models to be used in adults in the community to predict risk of development of chronic kidney disease in the future, and inform targeted screening and/or primary prevention |

## Search Terms and search strategy with full results

This search was adapted from *Poorthuis et al, Ammemwerth et al and Nadarajah et al*.^1-3^

Database(s): Ovid MEDLINE(R) ALL <1946 to January 31, 2024>

Search strategy:

| # | Searches | Results |
| --- | --- | --- |
| 1 | chronic kidney disease/ or chronic renal disease/ or chronic kidney failure/ or chronic renal failure/ or chronic kidney insufficiency/ or chronic renal insufficiency/ or CKD/ or CKF/ or CRF/ or CRD/ | 155909 |
| 2 | (chronic kidney disease or chronic renal disease or chronic kidney failure or chronic renal failure or chronic kidney insufficiency or chronic renal insufficiency or CKD or CKF or CRF or CRD).ti,ab. | 126503 |
| 3 | 1 or 2 | 217414 |
| 4 | ROC Curve/ or (stratification or discrimination or discriminate or c-statistic or c statistic or c-index or c index or Area under the curve or Calibration or Indices or Algorithm or Multivariable).ti,ab. | 1118356 |
| 5 | Mass screening/ or Screen*.ti,ab. | 1035559 |
| 6 | Prevalence/ or prevalenc*.ti,ab. or incidence/ or incidenc*.ti,ab. | 1881107 |
| 7 | population/ or population*.ti,ab. | 2232101 |
| 8 | 5 or 6 or 7 | 4436597 |
| 9 | (communit* or data*).ti,ab. | 5929670 |
| 10 | (general adj3 population).ti,ab. | 151740 |
| 11 | database/ or dataset/ | 2763 |
| 12 | (Electronic Health Record* or electronic medical record* or electronic personal record* or electronic patient record* or personal health record* or personal medical record* or computer health record* or computer medical record* or computer patient record* or ehr? or phr? or ephr? or emr? or paehr?).ti,ab. | 71573 |
| 13 | Electronic Health Records/ or exp medical records systems computerized/ or exp health records personal/ | 50950 |
| 14 | Primary Health Care/ or (primary care or general practic*).ti,ab. | 214730 |
| 15 | 9 or 10 or 11 or 12 or 13 or 14 | 6190498 |
| 16 | 3 and 4 and 8 and 15 | 2829 |
| 17 | limit 16 to english language | 2784 |

Database(s): Embase Classic+Embase <1947 to 2024 January 31>

Search Strategy:

| **#** | Searches | Results |
| --- | --- | --- |
| 1 | exp chronic kidney disease/ or exp chronic renal disease/ or exp chronic kidney failure/ or exp chronic renal failure/ or exp chronic kidney insufficiency/ or exp chronic renal insufficiency/ or exp CKD/ or exp CKF/ or exp CRF/ or exp CRD/ | 186325 |
| 2 | (chronic kidney disease or chronic renal disease or chronic kidney failure or chronic renal failure or chronic kidney insufficiency or chronic renal insufficiency or CKD or CKF or CRF or CRD).ti,ab,kw. | 217623 |
| 3 | 1 or 2 | 295203 |
| 4 | predict.ti. | 101783 |
| 5 | (validat* or rule*).ti,ab. | 1376229 |
| 6 | (predict* and (outcome* or risk* or model*)).ti,ab. | 1704765 |
| 7 | ((history or variable* or criteria or scor* or characteristic* or finding* or factor*) and (predict* or model* or decision* or identif* or prognos*)).ti,ab. | 5983857 |
| 8 | decision*.ti,ab. and statistical model/ | 8121 |
| 9 | (decision* and (model* or clinical*)).ti,ab. | 376064 |
| 10 | (prognostic and (history or variable* or criteria or scor* or characteristic* or finding* or factor* or model*)).ti,ab. | 470986 |
| 11 | (stratification or discrimination or discriminate or c statistic or c index or "area under the curve" or auc or calibration or indices or algorithm or multivariable).ti,ab. | 1549944 |
| 12 | receiver operating characteristic/ | 221392 |
| 13 | 4 or 5 or 6 or 7 or 8 or 9 or 10 or 11 or 12 | 8351645 |
| 14 | exp mass screening/ | 325091 |
| 15 | Screening.ab,ti,kw. | 990142 |
| 16 | exp prevalence/ | 1032692 |
| 17 | Prevalence.ab,ti,kw. | 1184941 |
| 18 | exp incidence/ | 707337 |
| 19 | Incidence.ab,ti,kw. | 1445267 |
| 20 | 14 or 15 or 16 or 17 or 18 or 19 | 3843611 |
| 21 | (communit* or data*).ti,ab. | 8181852 |
| 22 | (general adj3 population).ti,ab. | 227705 |
| 23 | database/ or dataset/ | 490044 |
| 24 | Electronic Health Records/ or (electronic health record* or electronic medical record* or electronic personal record* or electronic patient record* or personal health record* or personal medical record* or computer health record* or computer medical record* or computer patient record*).ti,ab. or (ehr? or phr? or ephr? or emr? or paehr?).ti,ab. | 145188 |
| 25 | 21 or 22 or 23 or 24 | 8493571 |
| 26 | 3 and 13 and 20 and 25 | 14142 |
| 27 | letter.pt. or letter/ | 1314677 |
| 28 | note.pt. | 975481 |
| 29 | conference abstract.pt. | 5041500 |
| 30 | editorial.pt. | 795581 |
| 31 | case report/ or case study/ | 3153657 |
| 32 | (letter or comment*).ti. | 250300 |
| 33 | 27 or 28 or 29 or 30 or 31 or 32 | 10634573 |
| 34 | animal/ not human/ | 1629959 |
| 35 | nonhuman/ | 7608771 |
| 36 | exp animal experiment/ | 3147361 |
| 37 | exp experimental animal/ | 873507 |
| 38 | animal model/ | 1760397 |
| 39 | exp rodent/ | 4554289 |
| 40 | (rat or rats or mouse or mice).ti. | 1834087 |
| 41 | 34 or 35 or 36 or 37 or 38 or 39 or 40 | 10532204 |
| 42 | 33 or 41 | 19880798 |
| 43 | 26 not 42 | 7798 |
| 44 | limit 43 to english language | 7530 |

## Inclusion and exclusion criteria pertaining to variables incorporated in models

In this review we were interested in models that could be used with data routinely-collected in the community to inform the individual risk of chronic kidney disease. To make the model useful it should not require additional visits for baseline risk stratification.

The information that was considered likely to be available in community-based settings were:

- Sociodemographic variables including but not limited to age, sex, and ethnicity.
- Disease conditions and procedures including but not limited to hypertension, diabetes mellitus, cardiovascular disease, renal stones and peripheral vascular disease.
- Clinical assessments including but not limited to heart rate, systolic and diastolic blood pressure, height, weight and body mass index.
- Medications prescribed including but not limited to antihypertensives, statins, antidepressants, anxiolytics/hypnotics and antipsychotics.
- Lifestyle factors including but not limited to smoking status and alcohol consumption.
- Simple laboratory tests and biomarkers including but not limited to total, high-density lipoprotein and low-density lipoprotein cholesterol, triglycerides, creatinine, c-reactive protein, erythrocyte sedimentation rate, urine albumin creatinine ratio.

We excluded the following types of variables that are not routinely available in community records, or are very rarely tested for in community clinical practice and so are not generalisable:

- Analysis of homocysteine, specific gravity and C-cystatin levels
- Analysis of renal ultrasound (USS) imaging
- Analysis of electrocardiograph (ECG) parameters
- Analysis of retinal photos

## PROBAST: Justifications for assessments for specific signalling questions

Each model was assessed for risk of bias as either “high”, “unclear” or “low” in four domains (participants, predictors, outcomes and analysis) through a range of signalling questions. Applicability to our review question was assessed for each model in three domains (participants, predictors and outcomes) using the same scale.^4^

Risk of bias

Domain 1: Participants

Signalling question 2: Were all inclusions and exclusions of participants appropriate?

We assessed risk of bias based on age cut-offs used for exclusion. The appropriateness of cut offs was decided by the expected incidence of chronic kidney disease in the general population based on available literature. The incidence rises significantly above 65 years, with the highest incidence above 85 years and a low incidence below 45 years.^5-6^ Thus overall, the consensus was that excluding patients below the age of 45 years would not exclude a subgroup that would alter the performance of the prediction model for the intended target population. In terms of an exclusion criteria at the top of the age stratum it was considered that excluding anyone below 85 years would be inappropriate based on a few reasons. First, incidence is high over 85 years;^5-6^ second, age has been frequently identified as an independent risk predictor for kidney disease.^6-7^ Thus if a study included the age range 45 – 85 years this contributed to the signalling question being marked as ‘Y’.

Domain 4: Analysis

Signalling question 4: Were participants with missing data handled appropriately?

We assessed risk of bias for missing data by assuming that if missing data was not mentioned at all that it was likely missing but not taken account for and thus marked as ‘N or PN’ and thus ‘high’ risk of bias. If there was any extent of missing data and methods had not been used to impute or an analysis had not been made to assess whether inclusion of missing values would have made a difference to performance measures, then the signalling question was marked as ‘N or PN’ and the overall domain as ‘high’ risk of bias.

## Excluded studies

**Studies excluded that met a number of inclusion criteria but utilised data not routinely available in a primary care setting.**

1. Sabanayagam C, Xu D, Ting DSW, Nusinovici S, Banu R, Hamzah H, et al. A deep learning algorithm to detect chronic kidney disease from retinal photographs in community-based populations. The Lancet Digital Health. 2020 Jun 1;2(6):e295–302.
2. Gu Y, Chen M, Zhu B, Pei X, Yong Z, Li X, et al. A risk scoring system for the decreased glomerular filtration rate in Chinese general population. Journal of Clinical Laboratory Analysis. 2020;34(4):e23143.
3. Zhao J, Zhang Y, Qiu J, Zhang X, Wei F, Feng J, et al. An early prediction model for chronic kidney disease. Sci Rep. 2022 Feb 17;12(1):2765.
4. Kuo CC, Chang CM, Liu KT, Lin WK, Chiang HY, Chung CW, et al. Automation of the kidney function prediction and classification through ultrasound-based kidney imaging using deep learning. npj Digit Med. 2019 Apr 26;2(1):1–9.
5. Ifraz GM, Rashid MH, Tazin T, Bourouis S, Khan MM. Comparative Analysis for Prediction of Kidney Disease Using Intelligent Machine Learning Methods. Computational and Mathematical Methods in Medicine. 2021 Dec 3;2021:e6141470.
6. Holmstrom L, Christensen M, Yuan N, Hughes JW, Theurer J, Jujjavarapu M, et al. Deep learning based electrocardiographic screening for chronic kidney disease [Internet]. medRxiv; 2022 [cited 2023 Jun 2]. p. 2022.03.01.22271473. Available from: <https://www.medrxiv.org/content/10.1101/2022.03.01.22271473v1>
7. Hao S, Fu T, Wu Q, Jin B, Zhu C, Hu Z, et al. Estimating One-Year Risk of Incident Chronic Kidney Disease: Retrospective Development and Validation Study Using Electronic Medical Record Data From the State of Maine. JMIR Medical Informatics. 2017 Jul 26;5(3):e7954.
8. Yuan Y, Yang L, Gao R, Chen C, Li M, Tang J, et al. Exploratory Study on Screening Chronic Renal Failure Based on Fourier Transform Infrared Spectroscopy and a Support Vector Machine Algorithm. Journal of Spectroscopy. 2020 Sep 9;2020:e7379242.
9. Luo W, Gong L, Chen X, Gao R, Peng B, Wang Y, et al. Lifestyle and chronic kidney disease: A machine learning modeling study. Frontiers in Nutrition [Internet]. 2022 [cited 2023 Jun 2];9. Available from: <https://www.frontiersin.org/articles/10.3389/fnut.2022.918576>

**Studies excluded that met a number of inclusion criteria but participant inclusion was restricted to an at risk population subset (e.g. those with a diagnosis of hypertension) such that they were not representative of the general population.**

1. Mocroft A, Lundgren J, Ross M, Law M, Reiss P, Kirk O, et al. A clinically useful risk-score for chronic kidney disease in HIV infection. Journal of the International AIDS Society. 2014;17(4S3):19514.
2. Ando M, Yanagisawa N, Ajisawa A, Tsuchiya K, Nitta K. A simple model for predicting incidence of chronic kidney disease in HIV-infected patients. Clin Exp Nephrol. 2011 Apr 1;15(2):242–7.
3. Libório AB, Barros RM, Esmeraldo RM, Oliveira MLMB, Silva GB, Daher EF. Creatinine-Based Equations Predicting Chronic Kidney Disease After Kidney Donation. Transplantation Proceedings. 2011 Sep 1;43(7):2481–6.
4. James MT, Pannu N, Hemmelgarn BR, Austin PC, Tan Z, McArthur E, et al. Derivation and External Validation of Prediction Models for Advanced Chronic Kidney Disease Following Acute Kidney Injury. JAMA. 2017 Nov 14;318(18):1787–97.
5. Mocroft A, Lundgren JD, Ross M, Law M, Reiss P, Kirk O, et al. Development and Validation of a Risk Score for Chronic Kidney Disease in HIV Infection Using Prospective Cohort Data from the D:A:D Study. PLOS Medicine. 2015 Mar 31;12(3):e1001809.
6. Gurudas S, Nugawela M, Prevost AT, Sathish T, Mathur R, Rafferty JM, et al. Development and validation of resource-driven risk prediction models for incident chronic kidney disease in type 2 diabetes. Sci Rep. 2021 Jul 1;11(1):13654.
7. Odubela OO, Odunukwe N, Peer N, Musa AZ, Salako BL, Kengne AP. Development and validation of risk models to predict chronic kidney disease among people living with HIV: protocol for a systematic review. BMJ Open. 2022 Jul 1;12(7):e061149.
8. Ngo TC, Hurley MP, Thong AE, Jeon SH, Leppert JT, Chung BI. Estimating the risk of chronic kidney disease after nephrectomy. Can J Urol. 2013 Dec;20(6):7035–41.
9. Li PI, Wang JN, Guo HR. Long-term quality-of-care score predicts incident chronic kidney disease in patients with type 2 diabetes. Nephrology Dialysis Transplantation. 2018 Nov 1;33(11):2012–9.
10. Zhang Q, Zhang J, Lei L, Liang H, Li Y, Lu J, et al. Nomogram to predict risk of incident chronic kidney disease in high-risk population of cardiovascular disease in China: community-based cohort study. BMJ Open. 2021 Nov 1;11(11):e047774.
11. Israni AK, Xiong H, Liu J, Salkowski N, Trotter JF, Snyder JJ, et al. Predicting End-Stage Renal Disease After Liver Transplant. American Journal of Transplantation. 2013 Jul 1;13(7):1782–92.
12. Hou J, Li J, Huang J, Lu C, Zhou J, Liu Y, et al. Relationship between the exposure to cumulative cardiovascular health behaviors and factors and chronic kidney disease—The Kailuan study. PLOS ONE. 2018 Aug 31;13(8):e0203171.
13. Dunkler D, Gao P, Lee SF, Heinze G, Clase CM, Tobe S, et al. Risk Prediction for Early CKD in Type 2 Diabetes. Clinical Journal of the American Society of Nephrology. 2015 Aug;10(8):1371.
14. Cao X, Yang B, Zhou J. Scoring model to predict risk of chronic kidney disease in Chinese health screening examinees with type 2 diabetes. Int Urol Nephrol. 2022 Jul 1;54(7):1629–39.
15. Hallan SI, Kwong D, Vikse BE, Stevens P. Use of a Prostate Symptom Score to Identify Men at Risk of Future Kidney Failure: Insights From the HUNT II Study. American Journal of Kidney Diseases. 2010 Sep 1;56(3):477–85.
16. Poda A, Kabore N, Malateste K, De Rekeneire N, Semde A, Bikinga Y, et al. Validation of the D:A:D chronic kidney disease risk score in people living with HIV: the IeDEA West Africa Cohort Collaboration. HIV Medicine. 2021;22(2):113–21.

**Studies excluded that met a number of inclusion criteria but the models were not derived and/or validated in community-based electronic health records**

1. Chien KL, Lin HJ, Lee BC, Hsu HC, Lee YT, Chen MF. A Prediction Model for the Risk of Incident Chronic Kidney Disease. The American Journal of Medicine. 2010 Sep 1;123(9):836-846.e2.
2. O’Seaghdha CM, Lyass A, Massaro JM, Meigs JB, Coresh J, D’Agostino RB, et al. A Risk Score for Chronic Kidney Disease in the General Population. The American Journal of Medicine. 2012 Mar 1;125(3):270–7.
3. Kshirsagar AV, Bang H, Bomback AS, Vupputuri S, Shoham DA, Kern LM, et al. A Simple Algorithm to Predict Incident Kidney Disease. Archives of Internal Medicine. 2008 Dec 8;168(22):2466–73.
4. Kwon KS, Bang H, Bomback AS, Koh DH, Yum JH, Lee JH, et al. A simple prediction score for kidney disease in the Korean population. Nephrology. 2012;17(3):278–84.
5. Thakkinstian A, Ingsathit A, Chaiprasert A, Rattanasiri S, Sangthawan P, Gojaseni P, et al. A simplified clinical prediction score of chronic kidney disease: A cross-sectional-survey study. BMC Nephrology. 2011 Sep 26;12(1):45.
6. Halbesma N, Jansen DF, Heymans MW, Stolk RP, de Jong PE, Gansevoort RT, et al. Development and Validation of a General Population Renal Risk Score. Clinical Journal of the American Society of Nephrology. 2011 Jul;6(7):1731.
7. Bradshaw C, Kondal D, Montez-Rath ME, Han J, Zheng Y, Shivashankar R, et al. Early detection of chronic kidney disease in low-income and middle-income countries: development and validation of a point-of-care screening strategy for India. BMJ Global Health. 2019 Sep 1;4(5):e001644.
8. Król E, Rutkowski B, Czarniak P, Kraszewska E, Lizakowski S, Szubert R, et al. Early Detection of Chronic Kidney Disease: Results of the PolNef Study. American Journal of Nephrology. 2008 Sep 23;29(3):264–73.
9. de Almeida EAF, Lavinas C, Teixeira C, Raimundo M, Nogueira C, João Melo M, et al. Evaluation of an Instrument for Screening Patients at Risk for Chronic Kidney Disease: Testing SCORED (Screening for Occult Renal Disease) in a Portuguese Population. Kidney and Blood Pressure Research. 2012 Aug 8;35(6):568–72.
10. Lee C, Yun HR, Joo YS, Lee S, Kim J, Nam KH, et al. Framingham risk score and risk of incident chronic kidney disease: A community-based prospective cohort study. Kidney Res Clin Pract. 2019 Mar 31;38(1):49–59.
11. Hippisley-Cox J, Coupland C. Predicting the risk of Chronic Kidney Disease in Men and Women in England and Wales: prospective derivation and external validation of the QKidney®Scores. BMC Family Practice. 2010 Jun 21;11(1):49.
12. Yu Y, Zhao Q, Jiang Y, Wang N, Liu X, Qiu Y, et al. Prediction models and nomograms of 3-year risk of chronic kidney disease in China: a study from the Shanghai Suburban Adult Cohort and Biobank (2016–2020). Annals of Translational Medicine. 2021 Nov;9(22):1690–1690.
13. Herget-Rosenthal S, Dehnen D, Kribben A, Quellmann T. Progressive chronic kidney disease in primary care: Modifiable risk factors and predictive model. Preventive Medicine. 2013 Oct 1;57(4):357–62.
14. Carrillo-Larco RM, Miranda JJ, Gilman RH, Medina-Lezama J, Chirinos-Pacheco JA, Muñoz-Retamozo PV, et al. Risk score for first-screening of prevalent undiagnosed chronic kidney disease in Peru: the CRONICAS-CKD risk score. BMC Nephrology. 2017 Nov 29;18(1):343.
15. Wen J, Hao J, Zhang Y, Cao K, Zhang X, Li J, et al. Risk scores for predicting incident chronic kidney disease among rural Chinese people: a village-based cohort study. BMC Nephrology. 2020 Apr 6;21(1):120.
16. Saranburut K, Vathesatogkit P, Thongmung N, Chittamma A, Vanavanan S, Tangstheanphan T, et al. Risk scores to predict decreased glomerular filtration rate at 10 years in an Asian general population. BMC Nephrology. 2017 Jul 17;18(1):240.
17. Umesawa M, Sairenchi T, Haruyama Y, Nagao M, Yamagishi K, Irie F, et al. Validity of a Risk Prediction Equation for CKD After 10 Years of Follow-up in a Japanese Population: The Ibaraki Prefectural Health Study. American Journal of Kidney Diseases. 2018 Jun 1;71(6):842–50.

## Bayesian meta-analysis of *c*-statistic / AUROC

All Bayesian meta-analysis models assume random effects by default. Results are based on the posterior median. Prediction intervals are directly obtained from the corresponding posterior quartiles. The standard model for random effects meta-analysis assumes that the ‘true’ performance is normally distributed within and across studies.^8^ Within-study normality of performance estimates can be justified with this selection of included studies because they are all large. *Snell et al.* showd that the between-study distribution of the *c-*statistic on the original scale is not normally distributed when there is variability in the predictor effect across studies (which is likely in this selection of studies as they include different populations, and adopt slightly different definitions for predictors).^8^ They found that the logit scale is more appropriate for the estimation of prediction interval. Consequently we used the “valmeta” function of the “metamisc” package in R software which applies a logit transformation to the *c-*statistic prior to calculation of summary *c-*statistic and prediction interval.^9^

For appropriate prior distributions we borrowed from earlier work by *Debray et al.* which recommended a half Student*-t* distribution with location *m*, scale σ, and *v* degrees of freedom where we set *m* = 0 and define σ equal to the largest empirical value of
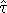
 (to allow for more extreme values of heterogeneity).^10^ These hyperparameter values allow to penalise the extent of between-study heterogeneity when the number of included validation studies is low.^10^ Further we also used *v* = 3 to ensure that the variance σ^2^ *v*/(*v*-2) exists and samples of τ were truncated above 10 to rule out unreasonable values. Thus the resulting priors are given as τdiscr ~ Student−*t*(0, 0.5^2^, 3)T[0.10] which has been shown to allow for large but realistic values for between-study heterogeneity.^10^

## GRADE: Standards used for judging criteria for downgrading and upgrading certainty of evidence

The certainty of the evidence was graded as ‘high’ (further research is very unlikely to change our confidence in the effect estimate), ‘moderate’ (further research is likely to have an important impact on our confidence in the effect estimate), ‘low’ (further research is very likely to have an important impact on our confidence in the effect estimate and is likely to change the estimate) or ‘very low’ (any estimate of effect is very uncertain).

The initial certainty level of the included prediction modelling studies was set at ‘high’ because the association between the predictors and outcomes was considered irrespective of any causal connection.^11^ Eight criteria were considered to further downgrade or upgrade the certainty of the evidence; five criteria which might downgrade the overall certainty of the evidence (methodological limitations of the study, indirectness, imprecision, inconsistency and likelihood of publication bias) and three which might potentially upgrade the overall certainty of the evidence (large effect, dose-response relation in the effect, and opposing plausible residual bias or confounding).

Methodological limitations of the studies were assessed by considering the overall risk of bias judgement across studies based on the overall PROBAST risk of bias assessment. Indirectness was assessed by making a global judgement on how dissimilar the research evidence is to the research question at hand (in terms of population and outcomes across studies).

Indirectness was assessed through concerns regarding the applicability of each included study from PROBAST (i.e. when the populations, predictors or outcomes of the study differ from the research question) and an overall judgement across studies was made.

Imprecision was assessed by considering the optimal total number of events across all studies. A minimum threshold of 10 events per variable was considered as the minimum required in regression modelling development studies, and 100 when machine learning methods had been used.^12,13^ For external validation studies a minimum sample size of at least 200 events was less concerning for imprecision.^14^ Results may also be imprecise when the 95% confidence intervals of c-statistic of all studies or of the largest studies include insufficient discrimination performance (0.5).

A global judgement on inconsistency was evaluated through the consistency of the model discrimination performance and the range of the 95% PI as a statistical measure of heterogeneity. Widely differing estimates of the c-statistic indicated inconsistency or if the 95% PI of the summary c-statistic was wide and included 0.5.

Publication bias was suspected when the body of evidence consisted of only positive studies from small sample sizes or all studies were funded by industry.

A large magnitude of effect (i.e. highly discriminatory predictive performance) was considered if the c-statistic exceeded 0.7 in the majority of studies.^15^ Since this review was not focused on drugs or pharmaceutical agents, assessing a dose-response gradient was not applicable here. Finally, we only included studies that described a multivariable prediction model and thus making a judgement whether all plausible confounders and biases were accounted for and may lead to an underestimated association is not applicable here.

# SUPPLEMENTARY RESULTS

## Studies included in systematic review and meta-analysis

1. Fraccaro P, van der Veer S, Brown B, Prosperi M, O’Donoghue D, Collins GS, et al. An external validation of models to predict the onset of chronic kidney disease using population-based electronic health records from Salford, UK. BMC Medicine. 2016 Jul 12;14(1):104.
2. Nelson RG, Grams ME, Ballew SH, Sang Y, Azizi F, Chadban SJ, et al. Development of Risk Prediction Equations for Incident Chronic Kidney Disease. JAMA. 2019 Dec 3;322(21):2104–14.
3. Stolpe S, Kowall B, Zwanziger D, Frank M, Jöckel KH, Erbel R, et al. External validation of six clinical models for prediction of chronic kidney disease in a German population. BMC Nephrology. 2022 Aug 1;23(1):272.
4. Collins G, Altman D. Predicting the risk of chronic kidney disease in the UK: an evaluation of QKidney® scores using a primary care database. Br J Gen Pract. 2012 Apr 1;62(597):e243–50.
5. Shih CC, Lu CJ, Chen GD, Chang CC. Risk Prediction for Early Chronic Kidney Disease: Results from an Adult Health Examination Program of 19,270 Individuals. International Journal of Environmental Research and Public Health. 2020 Jan;17(14):4973.
6. Bang H, Vupputuri S, Shoham DA, Klemmer PJ, Falk RJ, Mazumdar M, et al. SCreening for Occult REnal Disease (SCORED): A Simple Prediction Model for Chronic Kidney Disease. Archives of Internal Medicine. 2007 Feb 26;167(4):374–81.
7. Perez-Monteoliva NRR, Felix FJ, Lozano L, Miranda I, Fernandez-Berges D, Macías JF. The H.U.G.E. formula (hematocrit, urea, sex) for screening chronic kidney disease (CKD) in an age-stratified general population. J Nutr Health Aging. 2015 Jun 1;19(6):688–92.

## Supplementary Table S1: Outcomes of studies reporting on prediction models

| **Prediction Model** | **Study aim** | **Study (cohort)** | **CKD cases/total patients (%)** | **Discrimination** | | **Follow up duration (years)** | **ROB participants domain** | **ROB overall** |
| --- | --- | --- | --- | --- | --- | --- | --- | --- |
|  |  |  |  | ***c*-statistic** | **95% CI** |  |  |  |
| CKD-PC | EV | Nelson 2019 (OptumLabs1) | 12,427/113,481 (10.95) | 0.817 | 0.813 - 0.820 | 4 | Low | High |
| CKD-PC | EV | Nelson 2019 (OptumLabs2) | 7,891/172,858 (4.57) | 0.876 | 0.873 - 0.880 | 4 | Low | High |
| CKD-PC | EV | Nelson 2019 (OptumLabs3) | 8,482/131,345 (6.46) | 0.840 | 0.835 - 0.844 | 4 | Low | High |
| CKD-PC | EV | Nelson 2019 (OptumLabs4) | 34,049/399,325 (8.53) | 0.835 | 0.833 - 0.837 | 4 | Low | High |
| CKD-PC | EV | Nelson 2019 (OptumLabs5) | 9,393/91,287 (10.29) | 0.823 | 0.820 - 0.827 | 4 | Low | High |
| CKD-PC | EV | Nelson 2019 (OptumLabs6) | 6,569/90,284 (7.28) | 0.869 | 0.864 - 0.873 | 4 | Low | High |
| CKD-PC | EV | Nelson 2019 (OptumLabs7) | 7,396/115,915 (6.38) | 0.833 | 0.829 - 0.837 | 4 | Low | High |
| CKD-PC | EV | Nelson 2019 (OptumLabs8) | 90,073/837,339 (10.76) | 0.840 | 0.839 - 0.841 | 4 | Low | High |
| CKD-PC | EV | Nelson 2019 (OptumLabs9) | 1,632/33,962 (4.81) | 0.883 | 0.875 - 0.890 | 4 | Low | High |
| SCORED | EV | Stolpe 2022 (HNR) | 360/4,185 (8.60) | 0.720 | 0.700 - 0.750 | - | Unclear | High |
| Modified SCORED | EV | Stolpe 2022 (HNR) | 360/4,185 (8.60) | 0.730 | 0.700 - 0.750 | - | Unclear | High |
| Kearns | EV | Stolpe 2022 (HNR) | 360/4,185 (8.60) | 0.730 | 0.710 - 0.760 | - | Unclear | High |
| Kshirsagar | EV | Stolpe 2022 (HNR) | 360/4,185 (8.60) | 0.710 | 0.690 - 0.740 | - | Unclear | High |
| Kwon | EV | Stolpe 2022 (HNR) | 360/4,185 (8.60) | 0.720 | 0.700 - 0.750 | - | Unclear | High |
| Thakkinstian | EV | Stolpe 2022 (HNR) | - | 0.670 | 0.640 - 0.700 | - | Unclear | High |
| SCORED | IV | Bang 2007 (NHANES) | 601/8,530 (7.05) | 0.880 | 0.860 - 0.900 | - | Low | High |
| SCORED | EV | Bang 2007 (ARIC) | 392/12,038 (3.26) | 0.710 | 0.684 - 0.735 | - | Unclear | High |
| SCORED | EV | Fraccaro 2016 (SIRC) | 6,038/162,653 (3.71) | 0.899 | 0.895 - 0.903 | 5 | Low | Low |
| Chien | EV | Fraccaro 2016 (SIRC) | 6,038/162,653 (3.71) | 0.898 | 0.895 - 0.901 | 5 | Low | Low |
| Qkidney | EV | Fraccaro 2016 (SIRC) | 6,038/162,653 (3.71) | 0.910 | 0.907 - 0.913 | 5 | Low | Low |
| Kshirsagar | EV | Fraccaro 2016 (SIRC) | 6,038/162,653 (3.71) | 0.896 | 0.892 - 0.900 | 5 | Low | Low |
| Kwon | EV | Fraccaro 2016 (SIRC) | 6,038/162,653 (3.71) | 0.899 | 0.895 - 0.902 | 5 | Low | Low |
| O'Seaghdha | EV | Fraccaro 2016 (SIRC) | 6,038/162,653 (3.71) | 0.907 | 0.904 - 0.911 | 5 | Low | Low |
| Thakkinstian | EV | Fraccaro 2016 (SIRC) | 6,038/162,653 (3.71) | 0.892 | 0.888 - 0.985 | 5 | Low | Low |
| Qkidney | EV | Collins 2012 (THIN) | 43,186/1,593,506 (2.7) | 0.892 | 0.891 to 0.893 | 5 | Low | Unclear |
| Shih C4.5 | D and IV | Shih 2020 (Taiwan clinics) | 5,101/19,270 (26.5) | 0.788 | 0.781 – 0.795 | - | Low | Unclear |
| HUGE formula | EV | Perez-Monteoliva 2015 (HERMEX) | 62/2,831 (2.19) | 0.789 | 0.740 - 0.838 | - | Low | Low |

D, derivation; EV, external validation; IV, internal validation

## Supplementary Table S2: Calibration performance of models

| **Model** | **Study (cohort)** | **Calibration** | | | |
| --- | --- | --- | --- | --- | --- |
|  |  | **O:E ratio** | **Calibration slope (CI)** | **MAPE (SD)** | **Calibration plot reported** |
| CKD-PC | Nelson 2019 (OptumLabs1) | 0.868 | 0.920 (0.84, 0.99) | N/A | Yes |
| CKD-PC | Nelson 2019 (OptumLabs2) | 0.757 | 0.730 (0.7, 0.77) | N/A | Yes |
| CKD-PC | Nelson 2019 (OptumLabs3) | 0.924 | 0.930 (0.89, 0.96) | N/A | Yes |
| CKD-PC | Nelson 2019 (OptumLabs4) | 0.921 | 0.960 (0.89, 1.03) | N/A | Yes |
| CKD-PC | Nelson 2019 (OptumLabs5) | 0.947 | 0.990 (0.93, 1.06) | N/A | Yes |
| CKD-PC | Nelson 2019 (OptumLabs6) | 1.000 | 1.020 (0.96, 1.06) | N/A | Yes |
| CKD-PC | Nelson 2019 (OptumLabs7) | 0.976 | 0.980 (0.94, 1.03) | N/A | Yes |
| CKD-PC | Nelson 2019 (OptumLabs8) | 0.960 | 1.010 (0.93, 1.1) | N/A | Yes |
| CKD-PC | Nelson 2019 (OptumLabs9) | 0.784 | 0.770 (0.73, 0.8) | N/A | Yes |
| SCORED | Stolpe 2022 (HNR) | N/A | N/A | N/A | Yes |
| Modified SCORED | Stolpe 2022 (HNR) | N/A | N/A | N/A | Yes |
| Kearns | Stolpe 2022 (HNR) | N/A | N/A | N/A | Yes |
| Kshirsagar | Stolpe 2022 (HNR) | N/A | N/A | N/A | Yes |
| Kwon | Stolpe 2022 (HNR) | N/A | N/A | N/A | Yes |
| Thakkinstian | Stolpe 2022 (HNR) | N/A | N/A | N/A | Yes |
| SCORED | Bang 2007 (NHANES) | N/A | N/A | N/A | No |
| SCORED | Bang 2007 (ARIC) | N/A | N/A | N/A | No |
| SCORED | Fraccaro 2016 (SIRC) | N/A | 0.97 (0.96–0.98) | 0.063 (0.162) | No |
| Chien et al. | Fraccaro 2016 (SIRC) | N/A | 0.65 (0.64–0.65) | 0.081 (0.162) | No |
| Qkidney | Fraccaro 2016 (SIRC) | N/A | 1.02 (1.01–1.04) | 0.05 (0.166) | No |
| Kshirsagar | Fraccaro 2016 (SIRC) | N/A | 1.74 (1.72–1.76) | 0.068 (0.164) | No |
| Kwon | Fraccaro 2016 (SIRC) | N/A | 0.68 (0.67–0.69) | 0.086 (0.158) | No |
| O'Seaghdha | Fraccaro 2016 (SIRC) | N/A | 0.53 (0.52–0.53) | 0.089 (0.169) | No |
| Thakkinstian | Fraccaro 2016 (SIRC) | N/A | 0.44 (0.43–0.45) | 0.179 (0.161) | No |
| Qkidney | Collins 2012 (THIN) | N/A | N/A | N/A | Yes |
| Shih C4.5 | Shih 2020 (Taiwan clinics) | N/A | N/A | N/A | No |
| HUGE formula | Perez-Monteoliva 2015 (HERMEX) | N/A | N/A | N/A | No |

## Supplementary Table S3: Performance of machine learning and traditional regression techniques during model development

| **Author** | **Technique** | **Discrimination** | | **Calibration** | |
| --- | --- | --- | --- | --- | --- |
|  |  | ***c*-statistic** | **95% CI** | **p-value of GOF test** | **O:E ratio** |
| Shih 2020 | Classification and Regression Tree (CART) | 0.779 | 0.772 – 0.786 | N/A | N/A |
| Shih 2020 | Extreme Learning Machine (ELM) | 0.692 | 0.684 - 0.700 | N/A | N/A |
| Shih 2020 | C4.5 | 0.788 | 0.781 – 0.795 | N/A | N/A |
| Shih 2020 | Linear Discriminant Analysis (LDA) | 0.773 | 0.765 – 0.780 | N/A | N/A |

## Supplementary Table S4: Cohorts and number of predictors

| **Model** | **Study** | **Number of predictors** | **Derivation EHR cohort (country)** | **External validation EHR cohort (country)** |
| --- | --- | --- | --- | --- |
| CKD-PC | Nelson et al. | 9 | - | OptumLabs (US) |
| SCORED | Bang et al. | 10 | NHANES (US) | ARIC (US)  HNR (Germany)  SIRC (UK) |
| SCORED-Modified | Bang et al. | 8 | - | HNR (Germany) |
| Chien et al. | Chien et al. | 6 | - | SIRC (UK) |
| Qkidney | Hippisley-Cox et al. | 13 | - | SIRC (UK) |
| Kshirsagar et al. | Kshirsagar et al. | 8 | - | SIRC (UK) |
| Kwon et al. | Kwon et al. | 7 | - | SIRC (UK)  HNR (Germany) |
| O'Seaghdha et al. | O'Seaghdha et al. | 3 | - | SIRC (UK) |
| Thakkinstian et al. | Thakkinstian et al. | 5 | - | SIRC (UK)  HNR (Germany) |
| Kearns et al. | Kearns at al. | 6 | - | HNR (Germany) |
| Shih - C4.5 | Shih et al. | 10 | Taiwan clinics (Taiwan) | - |
| HUGE formula | Peres-Monteoliva et al. | 4 | - | HERMEX (Spain) |

## Supplementary Table S5: Baseline variables used in regression models

| **Model** | **Predictors** | | | | |
| --- | --- | --- | --- | --- | --- |
|  | **Patient characteristics** | **Medical history** | **Physical measurements** | **Investigations** | **Other** |
| CKD-PC | Age, Ethnicity, Sex | Hypertension, CVD | BMI | eGFR, AUCR | Smoking |
| SCORED | Age, Sex | Hypertension, DM, PVD, Anaemia, CCF, CVD |  | HDL, AUCR |  |
| Modified SCORED | Age, Sex | Hypertension, DM, CCF, CVD |  | HDL, AUCR |  |
| Chien et al. | Age | DM, Stroke | BMI, DBP | eGFR |  |
| QKidney | Ethnicity | Hypertension, MI, Angina, PHTN, DM, PVD, CCF, CVD, Renal stones |  |  | Family history, Smoking, Socioeconomic status |
| Kshirsagar et al. | Age, Sex | Hypertension, DM, PVD, Anaemia, CCF, CVD |  |  |  |
| Kwon et al. | Age, Sex | Hypertension, DM, Anaemia, CVD |  | AUCR |  |
| O'Seaghdha et al. | Age | Hypertension, DM |  |  |  |
| Thakkinstian et al. | Age | Hypertension, DM, Renal stones |  |  |  |
| Kearns et al. | Age, Ethnicity, Sex | Hypertension, IHD, CCF |  |  |  |
| Peres-Monteoliva HUGE |  |  |  | Urea, eGFR, RBC, Microalbuminuria |  |

## Supplementary Table S6: Baseline variable categories used in machine learning models

| **Model** | **Shih CART** | **Shih ELM** | **Shih C4.5** | **Shih LDA** |
| --- | --- | --- | --- | --- |
| **Variable category** |  |  |  |  |
| Diagnoses |  |  |  |  |
| Diagnostics | X | X | X | X |
| Medications |  |  |  |  |
| Hospitalisation |  |  |  |  |
| Clinical features |  |  |  |  |
| Demographics | X | X | X | X |
| Vital signs |  |  |  |  |
| Treatments/procedures |  |  |  |  |

## Supplementary Table S7: Risk of bias and applicability assessment for each PROBAST domain

| **Study** | **Model** | **Aim** | **ROB Participants** | **ROB Predictors** | **ROB Outcomes** | **ROB Analysis** | **Applicability Participants** | **Applicability Predictors** | **Applicability Outcomes** | **ROB Overall** | **Applicability Overall** |
| --- | --- | --- | --- | --- | --- | --- | --- | --- | --- | --- | --- |
| Nelson 2019 | CKD-PC | EV |  |  |  |  |  |  |  |  |  |
| Nelson 2019 | CKD-PC | EV |  |  |  |  |  |  |  |  |  |
| Nelson 2019 | CKD-PC | EV |  |  |  |  |  |  |  |  |  |
| Nelson 2019 | CKD-PC | EV |  |  |  |  |  |  |  |  |  |
| Nelson 2019 | CKD-PC | EV |  |  |  |  |  |  |  |  |  |
| Nelson 2019 | CKD-PC | EV |  |  |  |  |  |  |  |  |  |
| Nelson 2019 | CKD-PC | EV |  |  |  |  |  |  |  |  |  |
| Nelson 2019 | CKD-PC | EV |  |  |  |  |  |  |  |  |  |
| Nelson 2019 | CKD-PC | EV |  |  |  |  |  |  |  |  |  |
| Stolpe 2022 | SCORED | EV |  |  |  |  |  |  |  |  |  |
| Stolpe 2022 | Modified SCORED | EV |  |  |  |  |  |  |  |  |  |
| Stolpe 2022 | Kearns | EV |  |  |  |  |  |  |  |  |  |
| Stolpe 2022 | Kshirsagar | EV |  |  |  |  |  |  |  |  |  |
| Stolpe 2022 | Kwon | EV |  |  |  |  |  |  |  |  |  |
| Stolpe 2022 | Thakkinstian | EV |  |  |  |  |  |  |  |  |  |
| Bang 2007 | SCORED | D |  |  |  |  |  |  |  |  |  |
| Bang 2007 | SCORED | EV |  |  |  |  |  |  |  |  |  |
| Fraccaro 2016 | SCORED | EV |  |  |  |  |  |  |  |  |  |
| Fraccaro 2016 | Chien et al. | EV |  |  |  |  |  |  |  |  |  |
| Fraccaro 2016 | Qkidney | EV |  |  |  |  |  |  |  |  |  |
| Fraccaro 2016 | Kshirsagar et al. | EV |  |  |  |  |  |  |  |  |  |
| Fraccaro 2016 | Kwon et al. | EV |  |  |  |  |  |  |  |  |  |
| Fraccaro 2016 | O'Seaghdha et al. | EV |  |  |  |  |  |  |  |  |  |
| Fraccaro 2016 | Thakkinstian et al. | EV |  |  |  |  |  |  |  |  |  |
| Collins 2012 | Qkidney | EV |  |  |  |  |  |  |  |  |  |
| Shih 2020 | C4.5 | D |  |  |  |  |  |  |  |  |  |
| Perez-Monteoliva et al. 2015 | HUGE formula | EV |  |  |  |  |  |  |  |  |  |

Supplementary Figure S1: Sensitivity analysis for prediction models restricted to studies with low risk of bias for participants domain of PROBAST


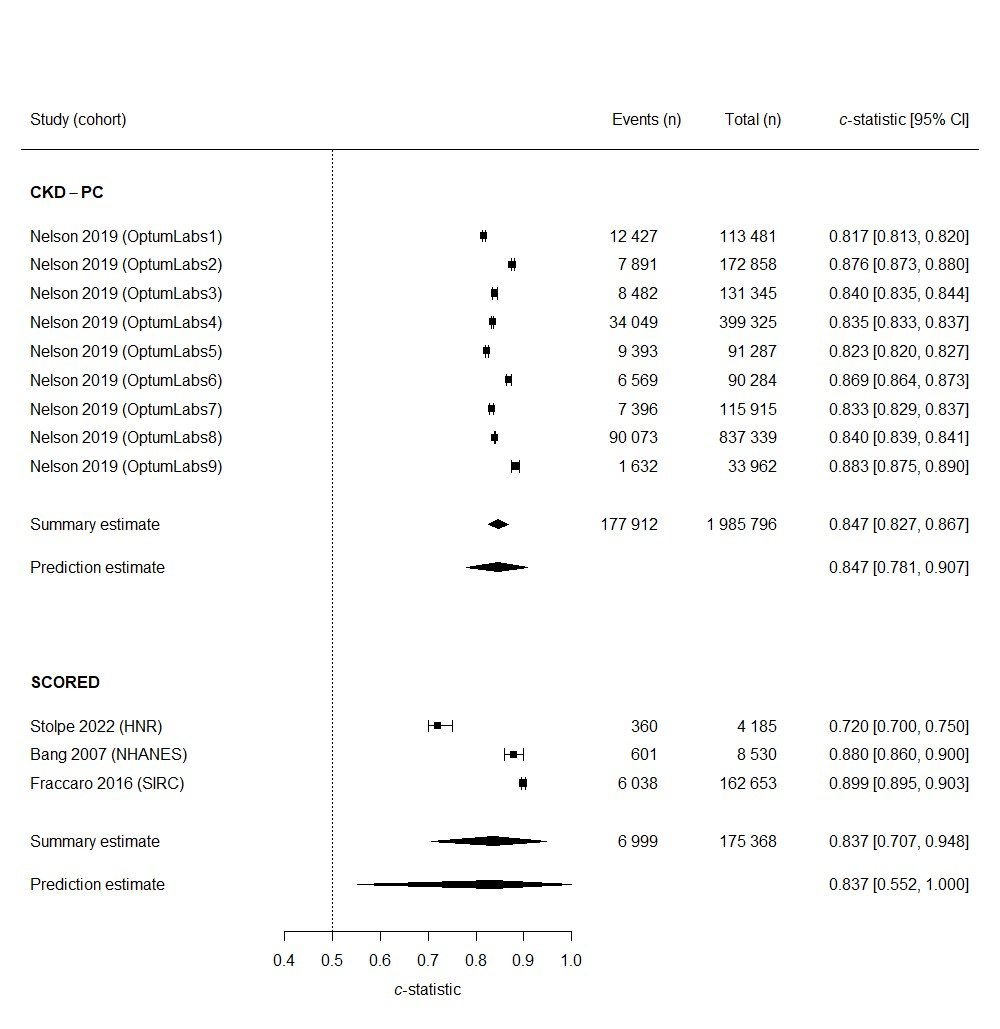


##

## Supplementary Figure S2: Sensitivity analysis for prediction models excluding results from development and internal validation


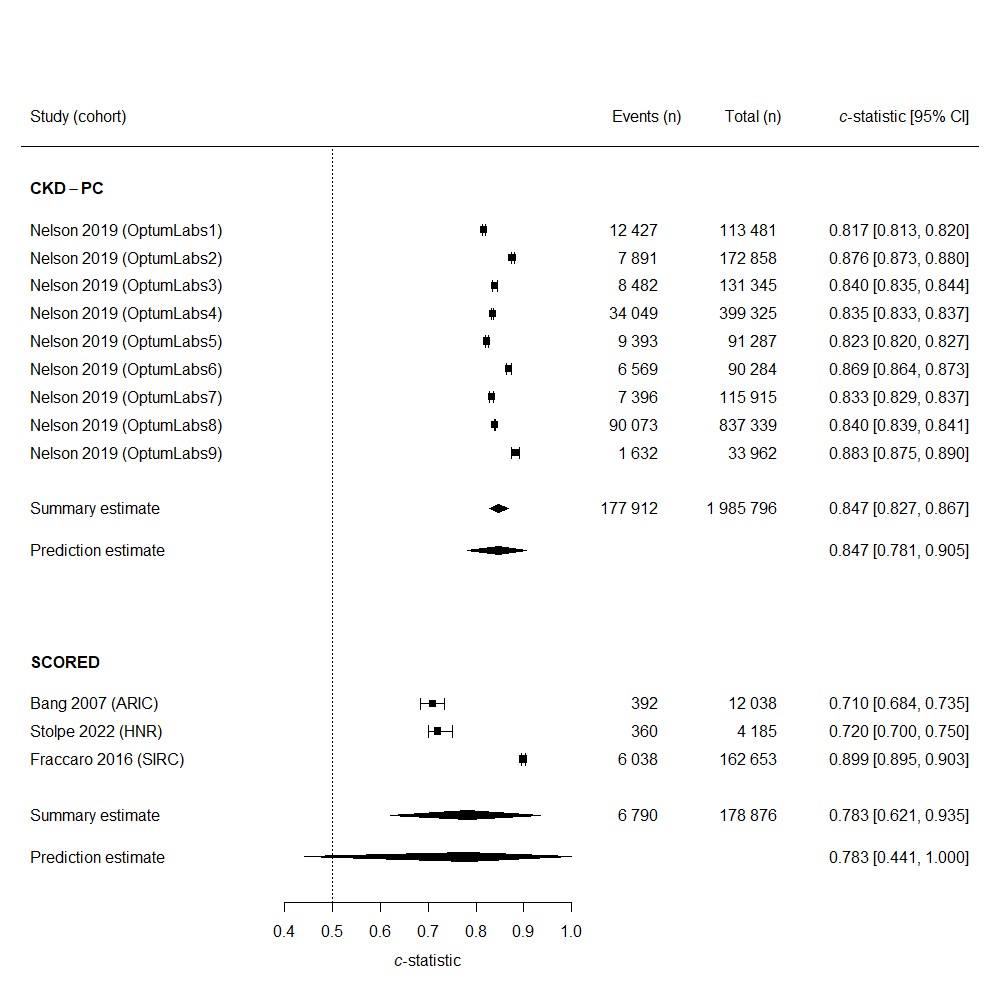


## Supplementary Figure S3: Funnel plot


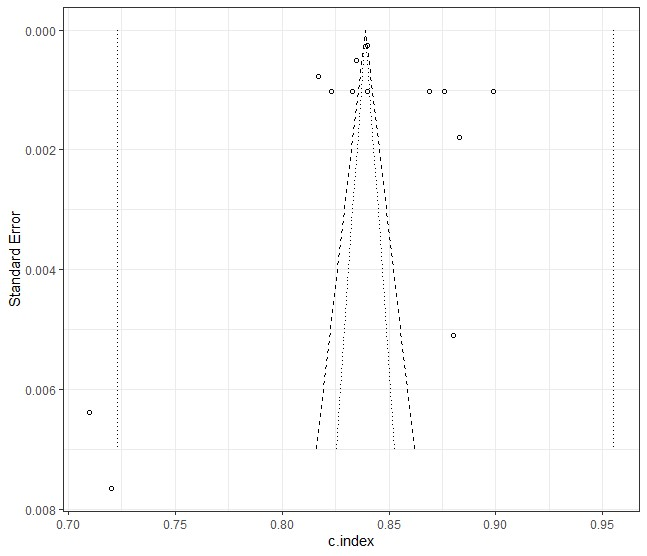


# PRISMA CHECKLIST

| **Section and Topic** | **Item #** | **Checklist item** | **Location where item is reported** |
| --- | --- | --- | --- |
| **TITLE** | | |  |
| Title | 1 | Identify the report as a systematic review. | 1 |
| **ABSTRACT** | | |  |
| Abstract | 2 | See the PRISMA 2020 for Abstracts checklist. | Abstract |
| **INTRODUCTION** | | |  |
| Rationale | 3 | Describe the rationale for the review in the context of existing knowledge. | 6-7 |
| Objectives | 4 | Provide an explicit statement of the objective(s) or question(s) the review addresses. | 7 |
| **METHODS** | | |  |
| Eligibility criteria | 5 | Specify the inclusion and exclusion criteria for the review and how studies were grouped for the syntheses. | 8 |
| Information sources | 6 | Specify all databases, registers, websites, organisations, reference lists and other sources searched or consulted to identify studies. Specify the date when each source was last searched or consulted. | 8-9, Supplementary material |
| Search strategy | 7 | Present the full search strategies for all databases, registers and websites, including any filters and limits used. | Supplementary material |
| Selection process | 8 | Specify the methods used to decide whether a study met the inclusion criteria of the review, including how many reviewers screened each record and each report retrieved, whether they worked independently, and if applicable, details of automation tools used in the process. | 8-9 |
| Data collection process | 9 | Specify the methods used to collect data from reports, including how many reviewers collected data from each report, whether they worked independently, any processes for obtaining or confirming data from study investigators, and if applicable, details of automation tools used in the process. | 9-10 |
| Data items | 10a | List and define all outcomes for which data were sought. Specify whether all results that were compatible with each outcome domain in each study were sought (e.g. for all measures, time points, analyses), and if not, the methods used to decide which results to collect. | 9-11 |
|  | 10b | List and define all other variables for which data were sought (e.g. participant and intervention characteristics, funding sources). Describe any assumptions made about any missing or unclear information. | 9 |
| Study risk of bias assessment | 11 | Specify the methods used to assess risk of bias in the included studies, including details of the tool(s) used, how many reviewers assessed each study and whether they worked independently, and if applicable, details of automation tools used in the process. | 10-11 |
| Effect measures | 12 | Specify for each outcome the effect measure(s) (e.g. risk ratio, mean difference) used in the synthesis or presentation of results. | 10 |
| Synthesis methods | 13a | Describe the processes used to decide which studies were eligible for each synthesis (e.g. tabulating the study intervention characteristics and comparing against the planned groups for each synthesis (item #5)). | 10-11 |
|  | 13b | Describe any methods required to prepare the data for presentation or synthesis, such as handling of missing summary statistics, or data conversions. | 10-11 |
|  | 13c | Describe any methods used to tabulate or visually display results of individual studies and syntheses. | 10-11 |
|  | 13d | Describe any methods used to synthesize results and provide a rationale for the choice(s). If meta-analysis was performed, describe the model(s), method(s) to identify the presence and extent of statistical heterogeneity, and software package(s) used. | 10-11 |
|  | 13e | Describe any methods used to explore possible causes of heterogeneity among study results (e.g. subgroup analysis, meta-regression). | 10-11 |
|  | 13f | Describe any sensitivity analyses conducted to assess robustness of the synthesized results. | 10-11 |
| Reporting bias assessment | 14 | Describe any methods used to assess risk of bias due to missing results in a synthesis (arising from reporting biases). | 10-11 |
| Certainty assessment | 15 | Describe any methods used to assess certainty (or confidence) in the body of evidence for an outcome. | 10-11 |
| **RESULTS** | | |  |
| Study selection | 16a | Describe the results of the search and selection process, from the number of records identified in the search to the number of studies included in the review, ideally using a flow diagram. | 12, Figure 1 |
|  | 16b | Cite studies that might appear to meet the inclusion criteria, but which were excluded, and explain why they were excluded. | Supplementary material |
| Study characteristics | 17 | Cite each included study and present its characteristics. | Table 1 |
| Risk of bias in studies | 18 | Present assessments of risk of bias for each included study. | Figure 3, Table S7 |
| Results of individual studies | 19 | For all outcomes, present, for each study: (a) summary statistics for each group (where appropriate) and (b) an effect estimate and its precision (e.g. confidence/credible interval), ideally using structured tables or plots. | 12, Table 1 |
| Results of syntheses | 20a | For each synthesis, briefly summarise the characteristics and risk of bias among contributing studies. | 12-13 |
|  | 20b | Present results of all statistical syntheses conducted. If meta-analysis was done, present for each the summary estimate and its precision (e.g. confidence/credible interval) and measures of statistical heterogeneity. If comparing groups, describe the direction of the effect. | 12-14, figure 4, S1, S2 |
|  | 20c | Present results of all investigations of possible causes of heterogeneity among study results. | N/A |
|  | 20d | Present results of all sensitivity analyses conducted to assess the robustness of the synthesized results. | Figure S1 |
| Reporting biases | 21 | Present assessments of risk of bias due to missing results (arising from reporting biases) for each synthesis assessed. | N/A |
| Certainty of evidence | 22 | Present assessments of certainty (or confidence) in the body of evidence for each outcome assessed. | 14 |
| **DISCUSSION** | | |  |
| Discussion | 23a | Provide a general interpretation of the results in the context of other evidence. | 15-18 |
|  | 23b | Discuss any limitations of the evidence included in the review. | 17-18 |
|  | 23c | Discuss any limitations of the review processes used. | 17-18 |
|  | 23d | Discuss implications of the results for practice, policy, and future research. | 15-17 |
| **OTHER INFORMATION** | | |  |
| Registration and protocol | 24a | Provide registration information for the review, including register name and registration number, or state that the review was not registered. | Abstract |
|  | 24b | Indicate where the review protocol can be accessed, or state that a protocol was not prepared. | Abstract |
|  | 24c | Describe and explain any amendments to information provided at registration or in the protocol. | Abstract |
| Support | 25 | Describe sources of financial or non-financial support for the review, and the role of the funders or sponsors in the review. | 20 |
| Competing interests | 26 | Declare any competing interests of review authors. | 20 |
| Availability of data, code and other materials | 27 | Report which of the following are publicly available and where they can be found: template data collection forms; data extracted from included studies; data used for all analyses; analytic code; any other materials used in the review. | 20 |

# PRISMA ABSTRACT CHECKLIST

| **Section and Topic** | **Item #** | **Checklist item** | **Reported (Yes/No)** |
| --- | --- | --- | --- |
| **TITLE** | | |  |
| Title | 1 | Identify the report as a systematic review. | Yes |
| **BACKGROUND** | | |  |
| Objectives | 2 | Provide an explicit statement of the main objective(s) or question(s) the review addresses. | Yes |
| **METHODS** | | |  |
| Eligibility criteria | 3 | Specify the inclusion and exclusion criteria for the review. | Yes |
| Information sources | 4 | Specify the information sources (e.g. databases, registers) used to identify studies and the date when each was last searched. | Yes |
| Risk of bias | 5 | Specify the methods used to assess risk of bias in the included studies. | Yes |
| Synthesis of results | 6 | Specify the methods used to present and synthesise results. | Yes |
| **RESULTS** | | |  |
| Included studies | 7 | Give the total number of included studies and participants and summarise relevant characteristics of studies. | Yes |
| Synthesis of results | 8 | Present results for main outcomes, preferably indicating the number of included studies and participants for each. If meta-analysis was done, report the summary estimate and confidence/credible interval. If comparing groups, indicate the direction of the effect (i.e. which group is favoured). | Yes |
| **DISCUSSION** | | |  |
| Limitations of evidence | 9 | Provide a brief summary of the limitations of the evidence included in the review (e.g. study risk of bias, inconsistency and imprecision). | Yes |
| Interpretation | 10 | Provide a general interpretation of the results and important implications. | Yes |
| **OTHER** | | |  |
| Funding | 11 | Specify the primary source of funding for the review. | Yes |
| Registration | 12 | Provide the register name and registration number. | Yes |

# REFERENCES

1. Ammenwerth E, Neyer S, Hörbst A, Mueller G, Siebert U, Schnell-Inderst PJ. Adult patient access to Ammenwerth E, Neyer S, Hörbst A, Mueller G, Siebert U, Schnell-Inderst PJ. Adult patient access to electronic health records. Cochrane Database of Systematic Reviews 2021(2).
2. Poorthuis MH, Jones NR, Sherliker P, Clack R, de Borst GJ, Clarke R, Lewington S, Halliday A, Bulbulia R. Utility of risk prediction models to detect atrial fibrillation in screened participants. European Journal of Preventive Cardiology 2020;**28**(6):586-595.
3. Nadarajah R, Alsaeed E, Hurdus B, Aktaa S, Hogg D, Bates MG, Cowan C, Wu J, Gale CP. Prediction of incident atrial fibrillation in community-based electronic health records: a systematic review with meta-analysis. Heart 2021.
4. Wolff RF, Moons KG, Riley RD, Whiting PF, Westwood M, Collins GS, Reitsma JB, Kleijnen J, Mallett S. PROBAST: a tool to assess the risk of bias and applicability of prediction model studies. Annals of internal medicine 2019;**170**(1):51-58.
5. Jonsson AJ, Lund SH, Eriksen BO, Palsson R, Indridason OS. Incidence of and risk factors of chronic kidney disease: results of a nationwide study in Iceland. Clinical Kidney Journal. 2022 Jul 1;15(7):1290–9.
6. Liu P, Quinn RR, Lam NN, Al-Wahsh H, Sood MM, Tangri N, et al. Progression and Regression of Chronic Kidney Disease by Age Among Adults in a Population-Based Cohort in Alberta, Canada. JAMA Network Open. 2021 Jun 8;4(6):e2112828.
7. Yamagata K, Ishida K, Sairenchi T, Takahashi H, Ohba S, Shiigai T, et al. Risk factors for chronic kidney disease in a community-based population: a 10-year follow-up study. Kidney International. 2007 Jan 2;71(2):159–66.
8. Snell KI, Ensor J, Debray TP, Moons KG, Riley RD. Meta-analysis of prediction model performance across multiple studies: Which scale helps ensure between-study normality for the C-statistic and calibration measures? Statistical Methods in Medical Research 2018;**27**(11):3505-3522.
9. Debray T, Jong Vd. metamisc: Meta-Analysis of Diagnosis and Prognosis Research Studies. In; 2020.
10. Debray TP, Damen JA, Riley RD, Snell K, Reitsma JB, Hooft L, Collins GS, Moons KG. A framework for meta-analysis of prediction model studies with binary and time-to-event outcomes. Statistical methods in medical research 2019;**28**(9):2768-2786.
11. Van Remoortel H, Scheers H, De Buck E, Haenen W, Vandekerckhove P. Prediction modelling studies for medical usage rates in mass gatherings: A systematic review. PLOS One 2020;**15**(6):e0234977.
12. Peduzzi P, Concato J, Feinstein AR, Holford TR. Importance of events per independent variable in proportional hazards regression analysis II. Accuracy and precision of regression estimates. Journal of clinical epidemiology 1995;**48**(12):1503-1510.
13. van der Ploeg T, Austin PC, Steyerberg EW. Modern modelling techniques are data hungry: a simulation study for predicting dichotomous endpoints. BMC medical research methodology 2014;**14**(1):1-13.
14. Collins GS, Ogundimu EO, Altman DG. Sample size considerations for the external validation of a multivariable prognostic model: a resampling study. Statistics in medicine 2016;**35**(2):214-226.
15. Pencina MJ, D’Agostino RB. Evaluating discrimination of risk prediction models: the C statistic. JAMA 2015;**314**(10):1063-1064.
